# Discriminating Between Living and Dead Bacteria With a Field‐Effect Transistor Based on Nano‐Channels

**DOI:** 10.1002/smll.74900

**Published:** 2026-07-28

**Authors:** Bar Chen, Vijay Garika, Shubham Babbar, Pooja Verma, Tom Cohen, Kfir Kattav, Fanny Akselrod, Aparnaa Shree Vijaikumar, Shankar Bhattarai, Sherina Harilal, Evgeny Pikhay, Inna Shechter, Doron Greental, Muhammad Y. Bashouti, Izhar Ron, Yakov Roizin, Barak Akabayov, Gil Shalev

**Affiliations:** ^1^ School of Electrical and computer Engineering Ben‐Gurion University of the Negev Beer‐Sheva Israel; ^2^ Department of Chemistry and Data Science Research Centre Ben‐Gurion University of the Negev Beer‐Sheva Israel; ^3^ Department of Solar Energy and Environmental Physics Swiss Institute for Dryland Environmental and Energy Research J. Blaustein Institutes for Desert Research Ben‐Gurion University of the Negev Midreshet Ben‐Gurion Israel; ^4^ Tower Semiconductor Migdal Haemek Israel; ^5^ The Ilse‐Katz Institute for Nanoscale Science and Technology Ben‐Gurion University of the Negev Beer‐Sheva Israel

**Keywords:** bacteria viability, *E. coli* sensing, FET biosensor, label‐free biosensing, real‐time bacterial detection, single‐cell analysis

## Abstract

Low‐cost and easy‐to‐operate biosensors allow for bacteria detection for on‐site and real‐time applications. However, current biosensors cannot distinguish between living and dead bacteria, critical in determining the persistence of bacterial contamination. The Meta‐Nano‐Channel field‐effect transistor (MNC FET) is employed. Its design allows the formation of numerous conducting nano‐channels each coupled to the sensing area binding events with a different efficiency addressing the challenge of molecular localized gating. The MNC FET is biofunctionalized with a recognition layer composed of *E. coli* antibodies. Sensing is performed in 0.5 µL drops of Lysogeny Broth (ionic strength of 197 mM, protein‐rich background of 200 mg/L), providing a complex food environment, and without either sample pre‐processing or pre‐measurement washing. Specific, label‐free, quantitative, and real‐time sensing is demonstrated with a limit‐of‐detection of a single‐bacterium and with excellent linearity and sensitivity. Furthermore, the platform's capacity to distinguish living from dead bacteria is validated by comparing the MNC FET response to living *E. coli* against populations subjected to thermal denaturation, antibiotic treatment, or metabolic inhibition. The MNC FET distinguishes between living and dead *E. coli* bacteria with a limit‐of‐detection of a single‐bacterium. This method paves the way for biosensors for the discrimination of bacterial viability.

## Graphical abstract

Bacterial viability is determined in a complex environment with a field‐effect biological transistor with a limit of detection of a single bacterium. Living, antibiotic‐treated, thermally denatured, and inhibited *E. coli* generate distinct electrical signatures, enabling label‐free single‐cell detection and providing new opportunities for rapid microbial diagnostics and phenotypic characterization.

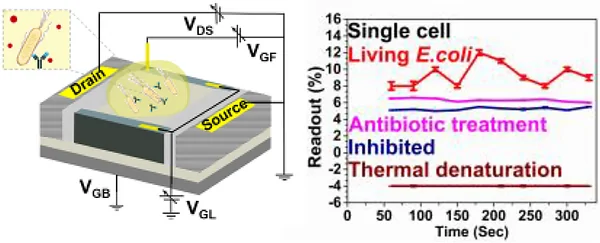

## 1 Introduction

Bacterial diseases rank among leading causes of morbidity and mortality worldwide [1, 2]. Foodborne diseases are caused by food contamination and occur at production, delivery, and consumption stages, encompassing a wide range of illnesses from diarrhea to cancer [3]. The World Health Organization (WHO) estimates 1.52 million deaths worldwide each year due to consumption of unsafe food [1, 2]. The ability for a rapid and accurate early detection of bacterial contamination in food is essential for preventing outbreaks of foodborne illnesses affecting millions of people globally [4, 5, 6]. Moreover, there is a pressing need to distinguish between dead and living bacteria in food. Living bacteria possess a full spectrum of physiological and structural properties that enable a cell to maintain its vitality: sustained metabolic activity, preservation of energy homeostasis, genomic replication and division, maintenance of cell envelope integrity, as well as dynamic traits such as motility and the ability to respond to physicochemical cues in the environment [7, 8]. In food safety, the ability to discriminate between living and dead bacteria is fundamental for evaluating product safety, quality, and probiotic functionality [9, 10]. Foodborne contamination is also a direct route by which living bacteria reach the human body, where viable cells can colonize tissues, secrete toxins, and cause infection [8, 11, 12]. In medical contexts, assessing bacterial activity is critical, since only living cells can initiate pathogenic processes, secrete toxins, proliferate and spread, and exhibit resistance to antimicrobial treatments. Also, detection of bacterial activity can support the prevention of antibiotic overuse, a phenomenon that leads to the development of antibiotic‐resistant bacteria [13]. From a biological and industrial perspective, viability provides essential insights into microbial population dynamics, their capacity for movement, adaptation to environmental changes, and maintenance of ecological interactions within complex environments [7, 11, 14]. In environmental and national security contexts, detection of bacteria activity is essential for accurate assessment of environmental contamination and prevention of infectious risk [15].

*E. coli* is a rod‐shaped Gram‐negative bacterium naturally residing in intestines of humans and other warm‐blooded animals. Most strains are harmless and play an essential role in digestion, helping to break down food and produce vital nutrients [16, 17]. However, pathogenic *E. coli*, Enterotoxigenic *E. coli* (ETEC) and Enteropathogenic *E. coli* (EPEC), can cause various illnesses [18, 19, 20], often associated with contaminated food, and due to its widespread presence and genetic flexibility, it is of a significant concern to public health [2]. Detecting the presence of *E. coli* is based on techniques such as cultural methods [21], biochemical tests [22], molecular methods [23], Immunological tests [24, 25], Microscopic methods [4, 26], all are either time consuming, expensive, or lack sufficient sensitivity. Moreover, discrimination between dead and living *E. coli* is critical for assessing infection risk, monitoring antibiotic effectiveness, and ensuring the microbiological safety of food and water. Traditional viability assays rely on culture‐based methods [11], fluorescence staining, viability PCR methods, and metabolic activity indicators all suffer from either long or complex processing inadequate for in‐situ applications [7, 11]. Hence, existing technologies fail to provide the need for real‐time accurate low‐cost in‐situ discrimination between living and dead *E. coli*.

Biological field‐effect transistors (FETs) offer quantitative, specific, label‐free, low‐cost, real‐time and potentially multiplexed sensing in small sample volumes [27]. The fundamental principle of operation of biological FETs is the transduction of electric fields, induced by biological interactions, into a significant variation in source‐drain current (*I_DS_*) [28, 29, 30, 31, 32, 33, 34, 35, 36, 37, 38]. Recent advancements in nanomaterial‐based platforms have pushed the boundaries of detection of dissolved gases [39, 40], biomarkers [41, 42, 43, 44], and microorganisms [45]. For instance, Feng et al. utilized aptamer‐functionalized carbon nanotube (CNT) FETs to achieve a single‐bacterium resolution for *Salmonella enterica* and *Staphylococcus aureus* in phosphate‐buffered saline (PBS) [31]. Similarly, Han and Jang integrated CNT FETs within microfluidic architectures for the continuous, label‐free monitoring of *S. aureus* in diluted PBS [46]. Beyond CNTs, graphene‐based FETs (G‐FETs) have demonstrated a comparable sensitivity; Kumar et al. leveraged dielectrophoresis to reach a detection limit of a single cell [47], while Tan et al. employed G‐FETs biofunctionalized with phage tail spike proteins to detect individual *E. coli* cells [48]. While these strategies predominantly rely on sophisticated recognition layers—such as aptamers or antibodies [30, 31, 34, 38, 46, 49]—to achieve high specificity, a critical gap remains. Despite the significant maturation of FET‐based diagnostics, the ability to distinguish between viable and nonviable cells remains an underexplored frontier in the current literature.

Living bacteria continuously alter their position, orientation, and local charge distribution due to their intrinsic motility and metabolic activity [50, 51, 52, 53]. The leading hypothesis of the study argues that the continuous activity of living bacteria can be detected by the mechanism of field‐effect transduction. In the current work, *E. coli* bacteria specifically bind with surface‐bound anti‐*E. coli* antibodies. Importantly, *E. coli* bacteria participate in a wide range of biological processes even after binding to surfaces [54, 55]. These processes include active motility via flagella, morphological changes, secretion of metabolites, and even cell division, all of which involve constant exchange of energy and charge with the environment [16, 56].

The current study demonstrates the Meta‐Nano‐Channel (MNC) FET for direct and label‐free discrimination between living and dead *E. coli*. Specifically, dead *E. coli* controls are established using three distinct mechanisms of inactivation: antibiotic treatment (kanamycin), thermal denaturation (heat‐killing), or metabolic inhibition. The MNC FET architecture is engineered to mitigate sensing limitations associated with solution potential control, as well as the stochastic nature of nonuniformly distributed molecular binding events [36, 37, 57, 58, 59, 60, 61, 62, 63, 64]. The sensing is performed in a complex medium of high ionic strength, providing real‐time discrimination between dead and living bacteria with a limit‐of‐detection (LOD) of a single bacterium. Finally, the sensing is performed without any sample pre‐processing and without the conventional pre‐measurement washing steps.

## 2 Methods

### 2.1 MNC FET Architecture, Fabrication, Principle of Operation

**FIGURE 1:**
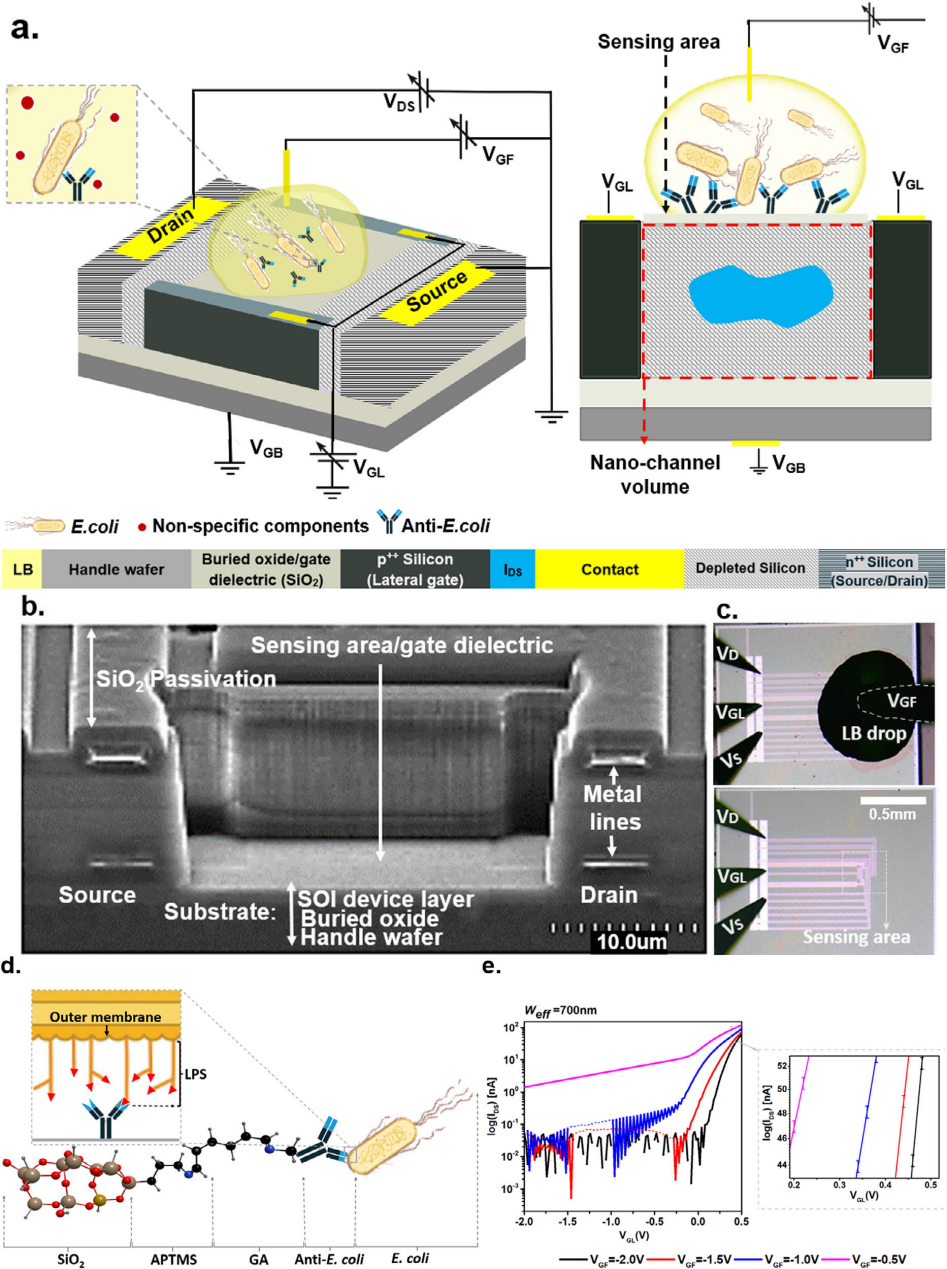
(a) A 3D illustration and a 2D illustrated cross‐section of the MNC FET. (b). Tilted SEM cross‐section of the MNC FET (before surface modification). (c). **Top**: An optical image of the MNC FET showing the 0.5 µL LB drop, electrical probes, and the quasi‐reference electrode. **Bottom**: An optical image of the MNC FET without the LB drop. (d). Illustration of the biofunctionalization process also showing the binding of the surface‐bound anti *E. coli*. receptor with *E. coli* LPS target. (e). *I_DS_–V_GL_* curves for selected *V_GF_* values for MNC FET biofunctionalized with anti‐*E. coli* antibodies. The measurements are performed for 0.5 µL LB drops. Data points and error bars are averages and standard deviations of 20 drops where each drop is measured three times.

Electron majority carriers form the 10 µm long conducting nano‐channel (*I_DS_*) electrically connecting the n‐type source and drain regions (accumulation mode conduction). The nano‐channel volume (See marked in 2D cross section of Figure 1a) is bound on the lateral sides by p‐type silicon regions forming lateral p‐n junctions (*V_GL_*). The current work employs two MNC FET architecture in which the distance between the p‐type regions is 700 nm and 900 nm (*W_eff_*). The source (*V_S_*), drain (*V_D_*), and p‐type regions are electrically contacted with aluminum lines. The back gate (*V_GB_*) and *V_S_* are grounded. MNC FET fabrication has been reported previously [36, 37, 57, 58, 59, 60, 61, 62, 63]. Briefly, the silicon part of the MNC biochip is fabricated (Tower Semiconductor Inc.) in a Complementary Metal‐Oxide‐Semiconductor (CMOS) flavored silicon‐on‐insulator (SOI) technology. The thickness of the SOI device layer and the buried oxide (BOX) are 145 nm and 400 nm, respectively. The device layer is n‐type doped to 10^17^ cm^−3^, the lateral p‐type regions are degenerated silicon, and the source and drain regions are degenerated n‐type. Standard CMOS self‐aligned silicidation is performed for all metal‐silicon contacts to ensure contacts of low ohmic resistance. The CMOS gate dielectric (sensing area) is 5.5 nm thermal oxide (SiO_2_) for low noise and efficient field‐effect transduction. The MNC FET is passivated with 1 µm of SiO_2_ except the electrical pads and the sensing area [46, 47]. Measurements of fabrication yield and variability are provided in Section S7. The MNC FET operates in the following manner: target molecules are introduced onto the sensing area (see Figure 1a for 2D cross‐section). The electric potential of the drop (*V_GF_*) is set by a commercial quasi‐reference electrode establishing boundary conditions for the Poisson and the Poisson‐Boltzmann equations determining the potential distributions in the conducting nano‐channel and the drop, respectively. Also, current at the quasi‐reference electrode is monitored to ensure negligible solution leakage current, enabling field‐effect transduction. *I_DS_* vs. *V_GL_* is measured for a constant *V_GF_*. Every *V_GL_* value determines different depletion widths at the lateral p‐n junctions, and hence a different size of *I_DS_*. Each *I_DS_* size, henceforth a nano‐channel, transduces binding events into *I_DS_* with a different efficiency [36, 37, 57, 58, 59, 60, 61, 62, 63]. Importantly, *I_DS_*‐*V_GL_* measurement is performed for a fixed *V_GF,_* forcing the solution and molecular species into electrochemical equilibrium during measurement [37, 60, 61].

### 2.2 Surface Modification and Characterization

Surface chemical modification is developed with 1 cm × 1 cm substrates of silicon decorated with 5.5 nm SiO_2_. The samples are sonicated in an ultrasonic bath (WULTSON, WSON‐32‐D, 40 kHz) for 2 min in ethyl acetate (Sigma‐Aldrich, 319902, ≥ 99.5%). This is repeated for 2‐propanol (Sigma‐Aldric, 190764, ≥ 99.5%), acetone (Sigma‐Aldric, 179973, ≥99.5%), and deionized water (DI). The samples are N_2_ dried after each sonication. Piranha solution is prepared with sulfuric acid (H_2_SO_4_, Sigma‐Aldrich, 339741, 98 wt.%) and hydrogen peroxide (H_2_O_2_, Sigma‐Aldrich, 1.08556, 35%) in a 3:1 ratio. The sensing area is incubated in piranha for 5 min, washed in DI water, and dried with N_2_. Then, the samples are incubated for 3 h in 3‐Amino‐Propyl‐Tri‐Methoxy‐Silane (APTMS, Sigma‐Aldrich, 946729, ≥ 99.9%) solution (0.1%V/V methanol). Then, the samples are rotated at 80 rpm (LW Scientific, RTL‐BLVD‐24T3) in methanol for three cycles, 3 min each. Then, the samples are submerged in DI water for 24 h to allow hydrolysis, dried with N_2_ and oven‐cured for 1 h at 120°C. Then, the samples are incubated for 40 min at 80 rpm in 0.5% Glutaraldehyde (GA, Sigma‐Aldrich, G5882, 50%), washed with 10 mM Phosphate‐buffered saline (PBS, Sigma‐Aldrich, P4417, pH 7.4) and dried with N_2_. Finally, the samples are incubated for 12 h in a 1 mg/mL of Anti‐*E. coli* (Abcam AB35654, Anti‐*E. coli* LPS antibody) in 0.1 mM PBS pH 7.4 (Sigma‐Aldrich, P4417, pH 7.4) at 4°C, then washed with 10 mM PBS and dried under N_2_. This chemical modification process is then applied to MNC biochips. A blocking step is performed following the immobilization of the recognition layer (control sample only). A blocking solution containing 1% Bovine Serum Albumin (BSA, Sigma‐Aldrich A7030, pH 7.4 ≥98%) in 1 mM PBS is prepared [65, 66, 67]. The BSA powder is vortex‐dissolved in PBS and applied onto the sensing area. The chips are incubated in BSA solution for 30 min at room temperature under static conditions. Then, the BSA solution is removed, and the surfaces are washed three times with PBS. The modifications are characterized by spectroscopic ellipsometry (Alpha‐SE Ellipsometer, J.A. Woollam), Electrochemical Impedance Spectroscopy (EIS) (Palmsens 4, Palmsens Inc.), contact angle tensiometer (Model OCA 20, dataphysics), and Atomic Force Microscopy (AFM, MFP‐3D‐Bio, Asylum Research/Oxford Instruments). The EIS measurements are conducted in 0.1 mM PBS pH 7.4. The sample is positioned within a three‐electrode electrochemical cell (0.8 mL). The back side of the silicon working electrode is scratched and applied with conductive carbon paint. A Pt wire (SEC‐C Pt Gauze working electrode, ALS Japan) serves as the counter electrode, and an Ag/AgCl electrode serves as the reference electrode (RE‐1BP, ALS Japan). To simulate physiological conditions, all AFM measurements are performed in 150 mM PBS at pH 7.4. Topographic images are acquired using a silicon cantilever probe (Olympus BL‐AC40TS‐C2) with a nominal tip radius of 9 ± 2 nm. The characterization data is presented in Section S1. Stability measurements of biofunctionalized MNC bioFET are provided in Section S7.

### 2.3 Preparation and Characterization of Bacteria

***E. coli***: *E. coli* DH5α is employed as target bacteria. Similar cultivation is performed for control *E. coli* BL21(DE3). Lysogeny Broth (LB, Difco, 244620) growth medium is prepared (Tryptone 10 g/L, Yeast extract 5 g/L, Sodium Chloride 10 g/L, Agar 15 g/L) and autoclaved. Starter culture of 10 µL glycerol stock of *E. coli* DH5α is added into 10 mL LB and incubated overnight at 37°C using an orbital shaker incubator (M.R.C. Ltd., LM‐570, 180 rpm). Eleven dilutions (henceforth ‘samples’) are prepared, and the absorbance of each is measured (Nanodrop 2000c Spectrophotometer, Thermo Scientific) at an optical density of 600 nm (OD_600_) to monitor *E. coli* growth until stationary phase is achieved (SI2). The bulk calibration is performed with LB. The samples are placed in ice. 100 µL from each sample is placed on LB agar plate (Difco, LB agar, Miller (Luria‐Bertani), DF0445076) to facilitate colony formation (Section S2). CFU/mL is determined after overnight incubation at 37°C (SI2), and OD_600_ vs. CFU/mL curve is established (see Section S2). *E. coli* cell number at the MNC FET sensing area is determined under an optical microscope at ambient conditions. The measurement is repeated four times for each sample, and the average and standard deviation are extracted (Section S4). ***Mycobacterium smegmatis* (*M. smegmatis*)**: 2.35 g of Middlebrook 7H9 broth powder (Difco, 2713100) is dissolved in 500 mL distilled water and autoclaved. Following cooling, the medium is supplemented with glycerol, dextrose, and Tween‐80 (Sigma‐Aldric, P6474, 0.05%). *M. smegmatis* cultures are initiated by adding 20 µL of glycerol stock (Sigma‐Aldric, P9769, ≥ 99.5%) into 10 mL supplemented 7H9 broth, followed by incubation at 37°C at 200 rpm for ∼48 h. The primary culture is vortexed and repeatedly pipetted. Serial ten‐fold dilutions are prepared by transferring 100 µL of culture into 900 µL of 7H9 medium. OD_600_ measurements are performed as described above. 5.7 g of Middlebrook 7H10 agar powder (Difco, 262710) is dissolved in 300 mL distilled water and autoclaved. Following cooling, the agar is supplemented with glycerol and dextrose (Sigma‐Aldric, PHR1000), poured into Petri dishes, and solidify. Dilutions are plated by spreading 100 µL of each dilution onto 7H10 agar plates. The plates are incubated at 37°C for 3–4 days, and CFU/mL is determined. **Antibiotic treatment**: Living *E. coli* DH5α samples are prepared as described above. The samples are subjected to 100 µg/mL kanamycin (CHEM‐IMPEX, 00195, ≥750 µg/mg (Potency)), incubated at 37°C for 2 h [76], centrifuged, the supernatant is removed, and the pellets are washed with LB. The pellets are resuspended in antibiotic‐free LB medium. The absence of living *E. coli* is verified by plating aliquots of the antibiotic‐treated samples onto LB agar plates (SI2). **Thermal denaturization**: Living *E. coli* DH5α samples are prepared as described above. The cultures are incubated until reaching OD_600_∼1.5, corresponding to a dense bacterial population in the late logarithmic growth phase. The samples are heated to 80°C for 20 min in water bath (ECOLINESTAREDITION, LAUDA E‐100). **Chloramphenicol Growth inhibition**: A 1 mg/mL chloramphenicol solution is prepared by diluting chloramphenicol (Fisher Scientific BioReagents, BP904‐100, >98%) in 100% ethanol from a 34 mg/mL stock solution. Nine tubes containing 5 mL LB are prepared with chloramphenicol concentrations ranging from 0 to 20 µg/mL. Then, 10 µL of overnight *E. coli* culture is added into each tube, followed by incubation at 37°C at 200–220 rpm with loosely closed caps for 4 h. OD_600_ is measured (Thermo Scientific, NanoDrop 2000c spectrophotometer). LB is supplemented with 1 µg/mL chloramphenicol followed by inoculation with overnight *E. coli* culture. Cultures are incubated at 37°C until reaching OD_600_ = 0.4–0.6, centrifuged, the supernatant is removed, and the pellet is resuspended in antibiotic‐free PBS. Then, the bacterial pellet is washed and temporarily resuspended in antibiotic‐free LB to remove residual chloramphenicol. **CFU determination and Bacterial viability test**: All above bacteria procedures are validated with CFU determination and viability test with the Resazurin Reduction Assay based on bacterial metabolic activity. See Sections S2 and S3 for details.

### 2.4 Background and Ferritin Controls

**Ferritin control**: Ferritin molecules (ABCAM, AB281293, >90%) are employed for a non‐specific control measurement. **Background control**: Bacterial cultivations and dilutions are performed according to the protocols described above. Bacterial suspensions are centrifuged at ∼14 000 rpm, the supernatant is collected and filtered with a 0.22 µm membrane filter (Millex, Durapore membrane, SLVV03RS). The filtration process removes intact bacterial cells while allowing soluble LB components, bacterial metabolites, residual proteins, and additional soluble biomolecular species to remain within the filtrate.

### 2.5 Electrical Measurements

**MNC FET**: The sensing measurements are performed for 0.5 µL drops of LB spiked with pre‐determined *E. coli* concentrations. The ionic strength of LB is ∼197 mM (Section S5 presents measurements for the determination of ionic strength of LB and *E. coli* solutions) with a background protein concentration of ∼0.2 mg/mL (See Section S6 for the determination of LB background protein concentration and *E. coli* solutions). The electrical pads of the MNC biochip are connected to Source Measuring Units (SMUs, B1500 Semiconductor Parametric Analyzer, Keysight Ltd.) with probe needles. The 0.5 µL LB drops are manually applied onto the MNC biochip with a pipette. The LB drop is spiked with an Ag/Ag^+^ quasi‐reference electrode for determination of drop potential. The Ag/Ag^+^ quasi‐reference electrode (connected to B1500 SMU) is formed from a commercial reference electrode (012171 RE‐7, ALS Co., Ltd), where the exposed Ag wire is decorated with an Ag/AgCl ink (011464, ALS Co., Ltd). Current‐voltage measurements are initiated 1 min after spiking the drop with the quasi‐electrode to allow the drop to reach electrochemical equilibrium. Each current‐voltage measurement lasts for 10 s, and a total of three successive current‐voltage measurements are performed for each drop (in‐drop measurement). Afterwards, the drop is removed with a dry clean‐room wipe, and the subsequent drop is applied and measured. For the time‐resolved measurements, each drop is measured 10 times. Finally, the highest *V_GL_* value is set to 0.5 V to avoid forward current across the lateral p‐n junctions for all *V_GF_* values. This measurement protocol is applied to all measurements of this study. **Conventional BioFET electrical measurements**: A conventional inversion‐mode hole‐channel biological FET configuration is realized with the same MNC FET. In this case the p‐type lateral gates function as source and drain, and the top part of the n‐type channel volume is inverted by applying negative potential to the solution electrode. The details are provided in Section S11.

### 2.6 3D Numerical Calculations

Numerical calculations are performed with Sentaurus Device by Synopsys. The details are provided in Section S10.

## 3 Results and Discussion

The MNC‐FET architecture is specifically designed to circumvent sensing bottlenecks, namely double‐layer dependency on solution potential and the dependency of sensing performance on the stochastic, non‐uniform distribution of analyte binding events. Figure 1a shows a three‐dimensional (3D) illustration of the MNC FET and a two‐dimensional (2D) generic cross‐section between source and drain. Figure 1b presents a Scanning Electron Microscopy (SEM) cross‐section of the MNC FET midway between source and drain, and Figure 1c presents an optical image of the MNC FET with and without the 0.5 µL LB drop. The quasi‐reference electrode and the electrical connections with probe needles are shown. Figure 1d illustrates the employed biofunctionalization following conventional amine‐based protocols [36, 37, 65, 66, 68, 69, 70] (see Methods). Briefly, the SiO_2_ sensing area is activated forming hydroxyl‐terminated surface for subsequent chemical coupling. The activated sensing area is chemically modified with APTMS, a silane compound with terminal amine groups, serving as reactive sites for conjugation. GA, a bifunctional cross‐linker, is then applied to covalently bridge the amine groups of APTMS to those on the antibody. Finally, an anti‐*E. coli* antibody is immobilized, enabling specific binding to *E. coli* targets. Physical surface characterization of the biofunctionalization is provided in Section S1.

Samples are introduced to the MNC FET via a standardized pipetting protocol (see Methods). Figure 1e presents *I_DS_*‐*V_GL_* curves for selected *V_GF_* values and for 0.5 µL LB drops applied onto an MNC FET (*W_eff_* = 700) biofunctionalized with anti‐*E. coli* antibodies. LB is chemically undefined, composed of peptides, amino acids, and other nutrients, like real food systems, where *E. coli* can thrive [16, 71, 72]. Each data point is an average of 20 drops, where each drop is measured 3 times, and the error bars are the respective standard deviations (see inset). The measurements commence 1 min after submerging the quasi‐reference electrode into the LB drop as during this time the measurements are not repeatable, indicating the drop has not reached electrochemical equilibrium. In contrast, note the excellent repeatability of measurements after 1 min, indicating the stability of the MNC FET with respect to random fluctuations in pH and electric potential of the LB drop. This set of 20 drops establishes the drop‐to‐drop variance and serves for the determination of the device's noise floor and resultant LOD. Also, the demonstrated drop‐to‐drop and in‐drop repeatability indicates that field‐effect transduction is immune to the continuous deposition of LB species on either the MNC FET sensing area and/or on the surface of the quasi‐reference electrode (this is further discussed below). *I_DS_* increases with increasing *V_GL_* as the depletion regions recede from the nano‐channel volume (forward bias of lateral p‐n junctions) and the effective nano‐channel width increases. Also,* I_DS_* increases with increasing *V_GF_* due to electron accumulation at the top part of the nano‐channel volume. Note that a unique combination of *V_GL_* and *V_GF_* defines a unique nano‐channel of specific location, size, and shape inside the nano‐channel volume. Finally, solution leakage current (*I_GF_*) is monitored during the measurements and its negligible value indicates the mechanism of field‐effect transduction rather than charge transport across the interface (see S7).

Label‐free sensing of living *E. coli* is pursued with the MNC FET. Figure 2a presents *I_DS_‐V_GL_* curves for selected* V_GF_* values and for *W_eff_* = 700 nm. Measurements are performed for 0.5 µL LB drops, spiked with different populations of living *E. coli* in the range of 0.75 ± 0.57 to 27.5 ± 1.3 (populations at MNC FET sensing area), which are introduced to the MNC FET sensing area (see Methods for the preparation of *E. coli* samples. See Sections S2, SI3 for validation and characterization of *E. coli* samples using orthogonal methods. See S4 for *E. coli* correlation curve between CFU/mL and *E. coli* cell number at the MNC FET sensing area.). The drops are applied and measured from the lowest *E. coli* population to the highest. Each data point is the average of three in‐drop measurements, and the error bars are the corresponding standard deviations (measurements commence 1 min after spiking the drop with the quasi‐reference electrode, as performed for Figure 1e). Figure 2b shows the respective normalized current, Readout (%) = 100 × (*I_E. coli_–I_LB_*)/*I_LB_*, where *I_E. coli_* and *I_LB_* are *I_DS_* values for drops containing *E. coli* and drops not containing *E. coli* (baseline), respectively. Readout increases with increasing *E. coli* population reflecting the accumulation of positive charge at the MNC FET sensing area (the mechanism is further discussed below). Importantly, the increase in current with the successive application of drops cannot be attributed to the buildup of non‐specific adsorption, as this option is excluded by the repeatability measurements of Figure 1e, which are performed in the exact same manner only with LB drops not containing *E. coli* (in either case the MNC FET is biofunctionalized with anti‐*E. coli* antibodies).

FIGURE 2(a) I_DS_–V_GL_ curves for selected values of V_GF_ and selected *E. coli* populations spiked into 0.5 µL LB drops (W_eff_ = 700 nm). The MNC FET is biofunctionalized with a recognition layer based on anti‐*E. coli* antibodies. Data points and error bars are in‐drop averages and standard deviations, respectively. (b). The corresponding Readout curves. (c). Illustrations of measurements with treated bacteria and controls.

**Figure:**
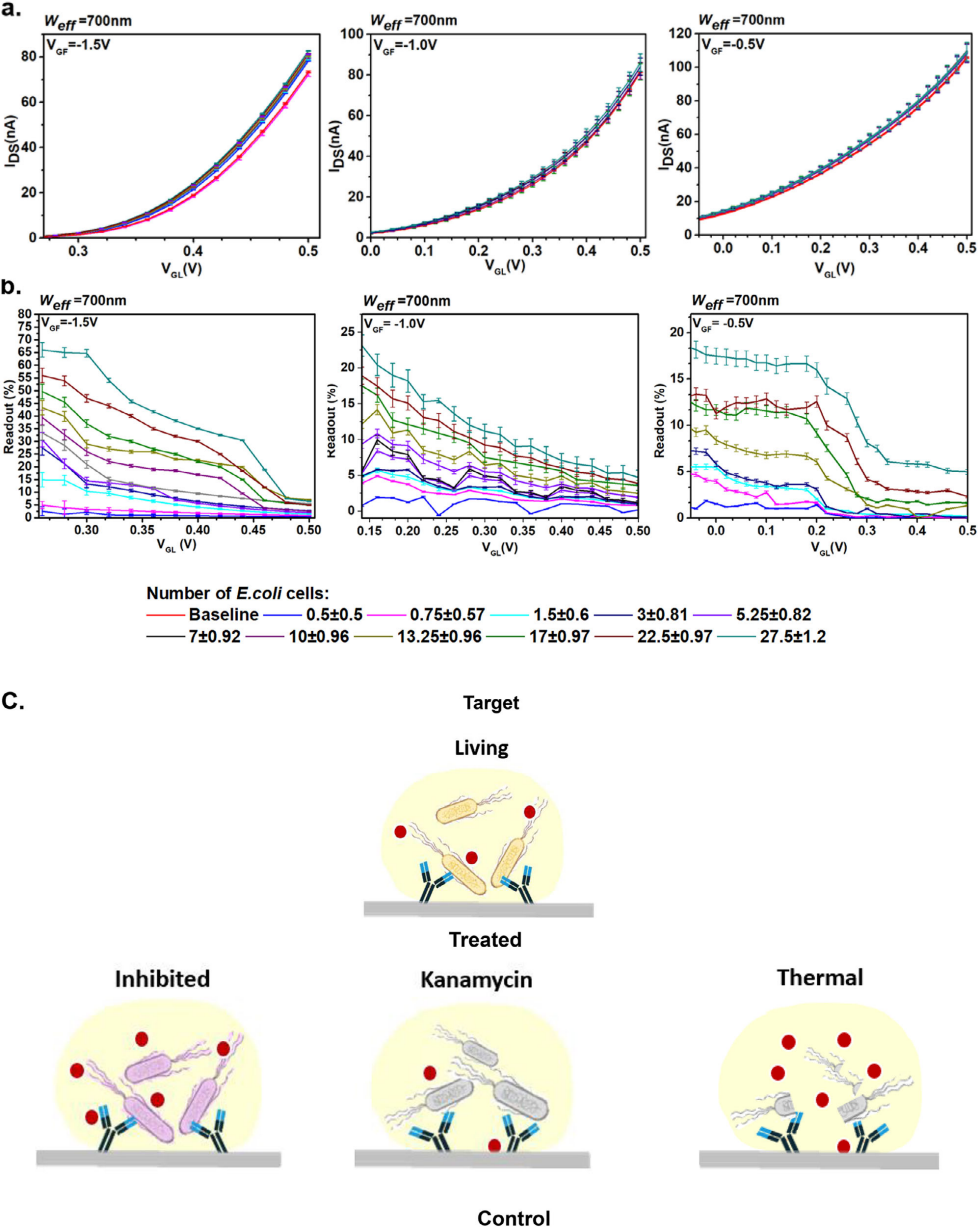

**Figure:**
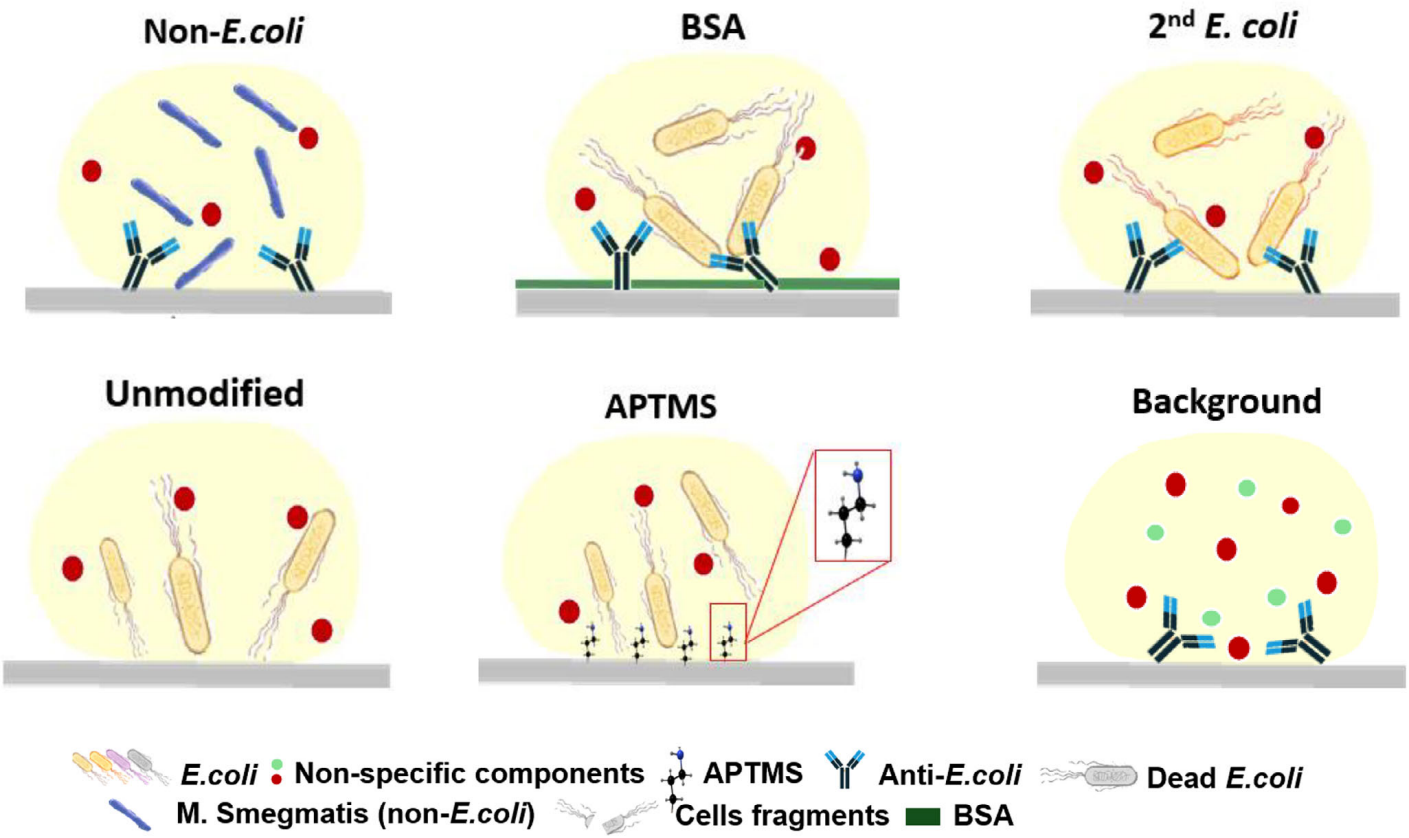

Similarly, the same measurements are repeated for 0.5 µL LB drops spiked with different populations of dead *E. coli* in the range of 0.75 ± 0.57 to 27.5 ± 1.3. Here, dead *E. coli* refers to bacteria treated in one of the following ways: (1) antibiotic treatment (kanamycin), (2) thermal denaturation, or (3) metabolic inhibition (see Methods). These treated bacteria are henceforth referred to as kanamycin, thermal and inhibited, respectively. (The validation of treated *E. coli* by orthogonal methods is provided in Sections S2 and S3, MNC FET *I_DS_*‐*V_GL_* curves of treated *E. coli* are provided in S7). Additional control measurements are performed to quantify the non‐specific signal. The control measurements follow the exact same procedure performed for Figure 2a. The control measurements are illustrated in Figure 2c: (1) introduction of 0.5 µL LB drops spiked with *E. coli* onto an unmodified MNC FET (henceforth, “unmodified” control), (2) introduction of 0.5 µL LB drops spiked with *E. coli* onto MNC FET modified with APTMS (henceforth, “APTMS” control), (3) introduction of 0.5 µL LB, after *E. coli* growth and after *E. coli* filtration, onto MNC FET modified with anti *E. coli* antibodies (henceforth, ‘background’ control), (4) introduction of 0.5 µL LB spiked with non‐*E. coli* bacteria *Mycobacterium smegmatis* to an MNC FET modified with anti *E. coli* antibodies (henceforth, “non‐*E. coli*” control) – this bacteria is selected due to the absence of cell envelope LPS, (5) introduction of 0.5 µL LB spiked with a different *E. coli* bacteria (*E. coli* BL21) to an MNC FET modified with anti *E. coli* antibodies (henceforth, “2nd *E. coli*” control), (5) introduction of 0.5 µL LB spiked with *E. coli* onto MNC FET modified with anti‐*E. coli* antibodies and blocked with BSA (henceforth, “BSA” control). The MNC FET *I_DS_*‐*V_GL_* curves and detailed explanations of control measurements are provided in S7.

**FIGURE 3:**
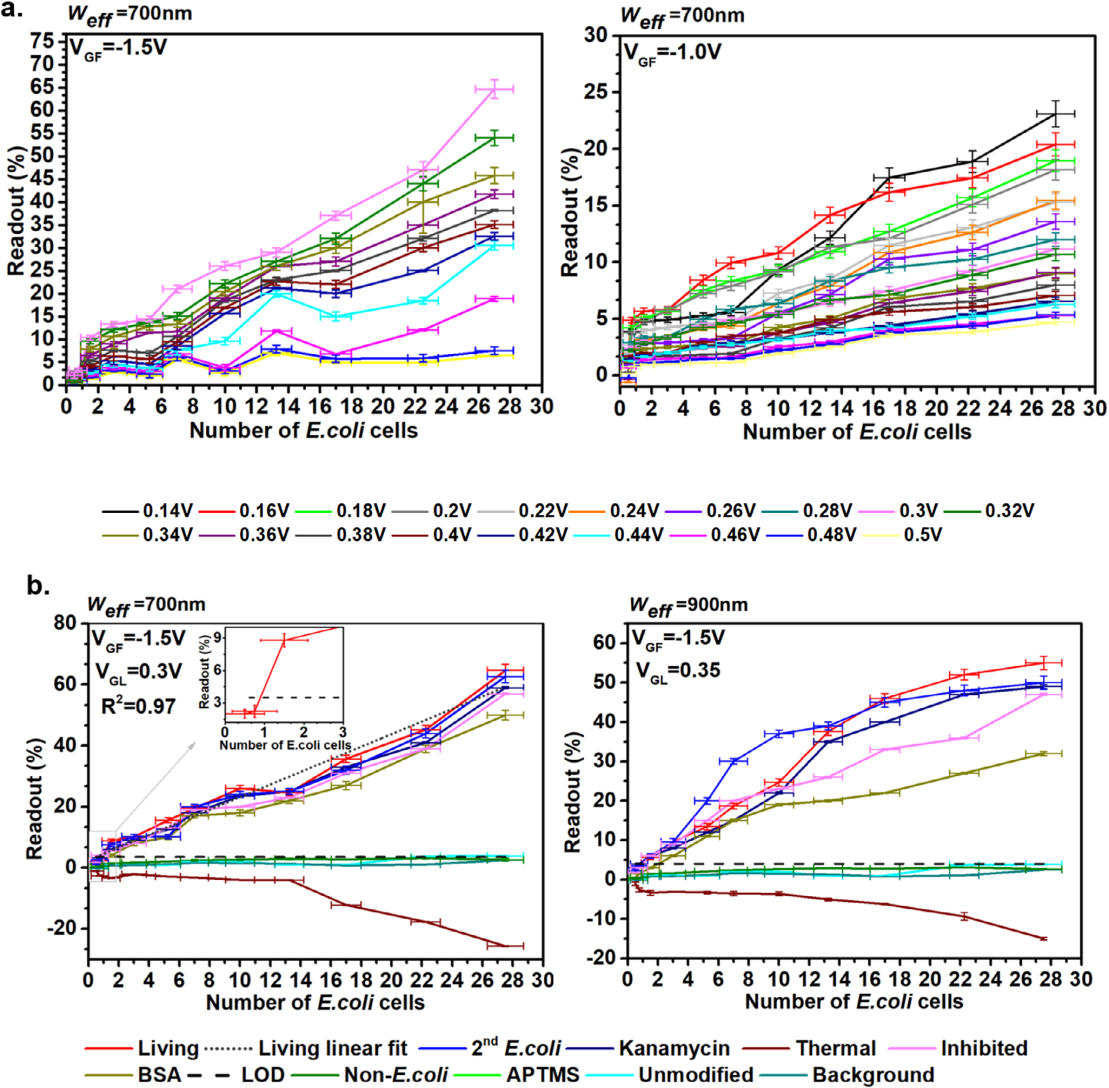
(a) Calibration curves for *W_eff_* = 700 nm and for *V_GF_* = −1.5 V, −1 V and selected *V_GL_* values. The data points and error bars are the averages and standard deviations, respectively, of three in‐drop measurements. The x‐axis error bars reflect the variation in *E. coli* population in the sensing area (SI4). (b) Calibration curves for specific sensing of living and treated *E. coli* for a selected nano‐channel configuration (*V_GF_* = −1.5 V, *V_GL_* = 0.3 V). The response curves of the control measurements are provided as well. The data points are the average values of two devices (*W_eff_* = 700 nm), and the y‐axis error bars are the respective standard deviations. Linear fit and R^2^ value are presented for the calibration curve of the living *E. coli* (DH5α). Also, following the *W_eff_* = 700 nm data, similar behavior is recorded for a single MNC FET with *W_eff_* = 900 nm. Here, the data points and error bars are the in‐drop averages and standard deviations, respectively.

The intersection of *V_GL_* and *V_GF_* defines a specific electrostatic configuration within the nano‐channel volume. Hence, *I_DS_*‐*V_GL_* characteristics at selected *V_GF_* values represent a discrete sampling of potential conductive paths. Because the distribution of binding events across the sensing area is inherently non‐uniform and stochastic, most conduction paths yield sub‐optimal transduction. However, through the systematic variation of gate potentials, specific nano‐channels can be identified *a posteriori* that possess the preferred spatial configuration to couple efficiently with the localized binding sites. Figure 3a displays calibration curves for living *E. coli* for *V_GF_* = −1,−1.5 V across a range of *V_GL_* values (*W_eff_* = 700 nm). Each data point reflects the average of three in‐drop measurements, with error bars denoting the in‐drop standard deviations. The x‐axis error bars reflect the error in determination of cell number at sensing area (Sections S2 and S4). The distinct sensitivity profiles—evidenced by the varying slopes of the calibration curves—underscore the necessity of this nano‐channel selection process to optimize field‐effect transduction. Notably, the enhancement in sensitivity observed at lower gate voltages suggests that selecting constricted, “nanoscopic” conduction paths is critical for maximizing the sensor's response to discrete biological targets. These experimental findings are corroborated by 3D numerical simulations, which demonstrate that a constricted conduction path enhances sensitivity toward localized surface charges (Section S10).

The specific, label‐free detection of *E. coli* is characterized by isolating the response of optimized nano‐channel configurations, with performance verified against the comprehensive suite of control experiments. Figure 3b shows calibration curves for the interaction of anti‐*E. coli* with both treated and living *E. coli* (*E. coli* DH5α and BL21) for a selected nano‐channel configuration of *V_GF_* = −1.5 V and *V_GL_* = 0.3 V (Sections S7–S9 provide calibration curves for additional conducting nano‐channels). The data points and error bars are the averages and standard deviations, respectively, of two identical devices (*W_eff_* = 700 nm). Control measurements are also presented, all of which demonstrate negligible responses with the exception of BSA control; the BSA control exhibits a signal profile similar to that of the unblocked devices, suggesting that non‐specific adsorption contributes negligibly to measurements performed without BSA blocking. Field‐effect transduction of non‐specific adsorption is discussed below. Finally, the LOD dashed line reflects the drop‐to‐drop variance extracted from Figure 1e. Figure 3b also presents the same only for a single device with *W_eff_* = 900 nm.

Note that the MNC FET responds to increasing population of living (*E. coli* DH5α and BL21), kanamycin‐treated, and inhibited bacteria in a similar manner. This is expected as anti‐*E. coli* antibodies do not discriminate between living and treated bacteria. As the outer membrane and LPS structures remain intact, the treated cells are still capable of binding the anti‐*E. coli* antibodies immobilized on the sensing area. *I_DS_* increase with increasing *E. coli* population irrevocably indicates the accumulation of positive charge at the sensing area upon the formation of anti‐*E. coli/E. coli* complexes. The response of the thermally denatured bacteria is entirely different; a decrease in current with increasing population indicates an accumulation of negative charge. This suggests that the interaction is inherently distinct from that of surface‐bound antibodies with intact bacteria (the proposed origins of positive and negative surface charges are discussed below).

The origin of *I_DS_* increase is discussed next. A FET biosensor translates biological events into electrical signals by the field‐effect phenomenon where electrostatic perturbation at the sensing area induces an electric field penetrating the electrically insulating gate dielectric and modifying *I_DS_* conductivity (via band bending). Biological events participating in the field‐effect are determined by the solution Debye length, extending from the sensing area into the bulk solution, which is inversely proportional to solution ionic strength [73]. Note that capacitive mechanisms governed by the Donnan potential are excluded, as discussed previously [58]. Debye screening length has been considered a major obstacle in the realization of FET‐based biosensors [35, 74, 75, 76, 77]. The Debye length of LB solution with an ionic strength of 197 mM is ∼0.7 nm (Section S5 presents measurements for ionic strength determination of LB, and the respective Debye length calculations) suggesting that the main contribution to the field‐effect is related to surface rather than bulk solution phenomena. Therefore, *I_DS_* increase with increasing *E. coli* population (treated and living), combined with the sub‐nanometer Debye length, indicates the accumulation of positive surface charge at the sensing area upon the formation of *E. coli*/Anti‐*E. coli* complexes (This is also supported by numerical data presented in Section S10). To further validate this surface charge accumulation, *E. coli* sensing is evaluated in 150, 1.5, and 0.15 mM PBS, corresponding to Debye lengths of 0.7, 10, and 30 nm, respectively. Debye lengths of 10 nm and 30 nm yield similar sensing performances, indicating that the interactions occur entirely within the Debye volume in both cases. Notably, a robust calibration curve is obtained at a Debye length of 0.7 nm; despite its lower sensitivity, this result supports the hypothesis that these interactions induce a significant surface charge (the corresponding data are presented in Section S5). Finally, AFM measurements are conducted for MNC FET biofunctionalized with anti‐*E. coli*. These measurements are performed in 150 mM PBS (pH 7.4) to mimic LB ionic strength. The extracted antibody height distribution reveals that most of the antibodies exhibit a height under 3 nm, which clearly indicates a tilt‐down orientation supporting the hypothesis that binding events occur in proximity to the sensing interface (see Section S1). The dominant tilt‐down orientation of surface adsorbed antibodies has been previously reported by others [78, 79, 80, 81].

Next, the origin of the positive surface charge is discussed. In general, isolated (before interacting with environment) *E. coli* is negatively charged due to surface membrane and LPS charges [82]. The phospholipid heads of the inner membrane are composed of negatively charged phosphate and carboxyl groups providing the membrane an overall negative charge, and the outer membrane contains LPS, which are also composed of negatively charged groups [15, 50, 51, 72, 82, 83, 84, 85, 86, 87, 88, 89, 90, 91]. Once *E. coli* is surrounded by an electrolyte, an electric double layer is formed to establish electrochemical equilibrium and to stabilizes the membrane [82, 91]. In the present case, the bacteria is surrounded by an LB medium containing high concentrations of inorganic ions such as Na^+^, K^+^, and divalent cations Mg^2^^+^ and Ca^2^^+^ [92], as well as positively charged organic molecules, short peptides and basic amino acids such as lysine, arginine, and histidine, polyamines such as putrescine and cadaverine, and yeast extract components with amino groups [92]. To account for *I_DS_* increase, it is suggested that monovalent and divalent cations partially neutralize the surface negative membrane and LPS charge [93, 94, 95, 96, 97, 98, 99]. Moreover, the formation of electric double layer does not only reduce or neutralize the *E. coli* negative surface charge but, at high cation concentrations, can induce a local ‘charge inversion’ where an excess layer of positive ions accumulates on the surface. Hence, in a nutrient‐rich environment like LB, with a complex mixture of divalent and monovalent cations alongside positively charged organic molecules, the bacterial surface can exhibit a positive surface charge [91, 92, 93], supporting Readout increase with increasing *E. coli* population. To corroborate the origin of the positive surface charge, *E. coli* sensing is also performed in 0.15, 1.5, and 150 mM PBS (Section S5). Interestingly, detection in PBS results in a current decrease, indicating an accumulation of negative surface charge. This contrasting behavior suggests that the positive response observed in LB may be attributed to its higher concentration of positive charges, as discussed above. Furthermore, the thermally denatured bacteria exhibit a current decrease, suggesting a fundamentally different electrostatic profile compared to the living, kanamycin, and inhibited bacteria. Nevertheless, the variations observed between the measurements taken in LB, those in PBS, and the denatured bacteria in LB highlight a mechanistic divergence that warrants further investigation.

Next, specificity and selectivity are considered. First, note the contrast between the calibration curves of living *E. coli*, kanamycin and inhibited and those of the control measurements, concluding that the anti‐*E. coli*/*E. coli* calibration curves reflect specific detection. Secondly, as predicted, the 2nd *E. coli* (*E. coli* BL21) performs similar to the target *E. coli* (DH5α) while a negligible response is recorded for the non‐*E. coli* control. Thirdly, the BSA control shows a similar response as living, kanamycin and inhibited bacteria suggesting that field‐effect transduction is not affected by non‐specific adsorption. Importantly, note that premeasurement washing steps are not performed and the drop is measured as applied (See Methods). It is assumed that the difference between the specific and non‐specific responses is traced to surface biomolecular organization. It is suggested that specific adsorption provides preferred surface molecular organization resulting in accumulation of surface charge, whereas non‐specific adsorption entails surface disorganization and prevents accumulation of net charge [69]. To validate these findings, non‐specific sensing control experiments are performed utilizing ferritin as a model protein (see Methods and Section S6). The measurements follow the exact procedure established for the unmodified control (specific sensing of ferritin with the MNC FET has been demonstrated [60]). Measurements across a range of ionic strengths (0.15, 1.5, and 150 mM PBS) consistently yield a null response. With that said, surface organization of biomolecular complexes cannot be ascertained with field‐effect devices, and the validation of the proposed hypothesis is left for future research. Finally, the performance for specific and label‐free sensing of living *E. coli* is extracted, for the calibration curve presented in Figure 3 (W_eff_ = 700 nm), with LOD of single bacterium, a dynamic range extending from 1.5 ± 0.6 to 27.5 ± 1.2 *E. coli*, a sensitivity of 2.06 ± 0.14%Readout/cell (slope of calibration curve), and a linearity of R^2^ = 0.97 (See Section S12 for additional statistical analysis).

**FIGURE 4:**
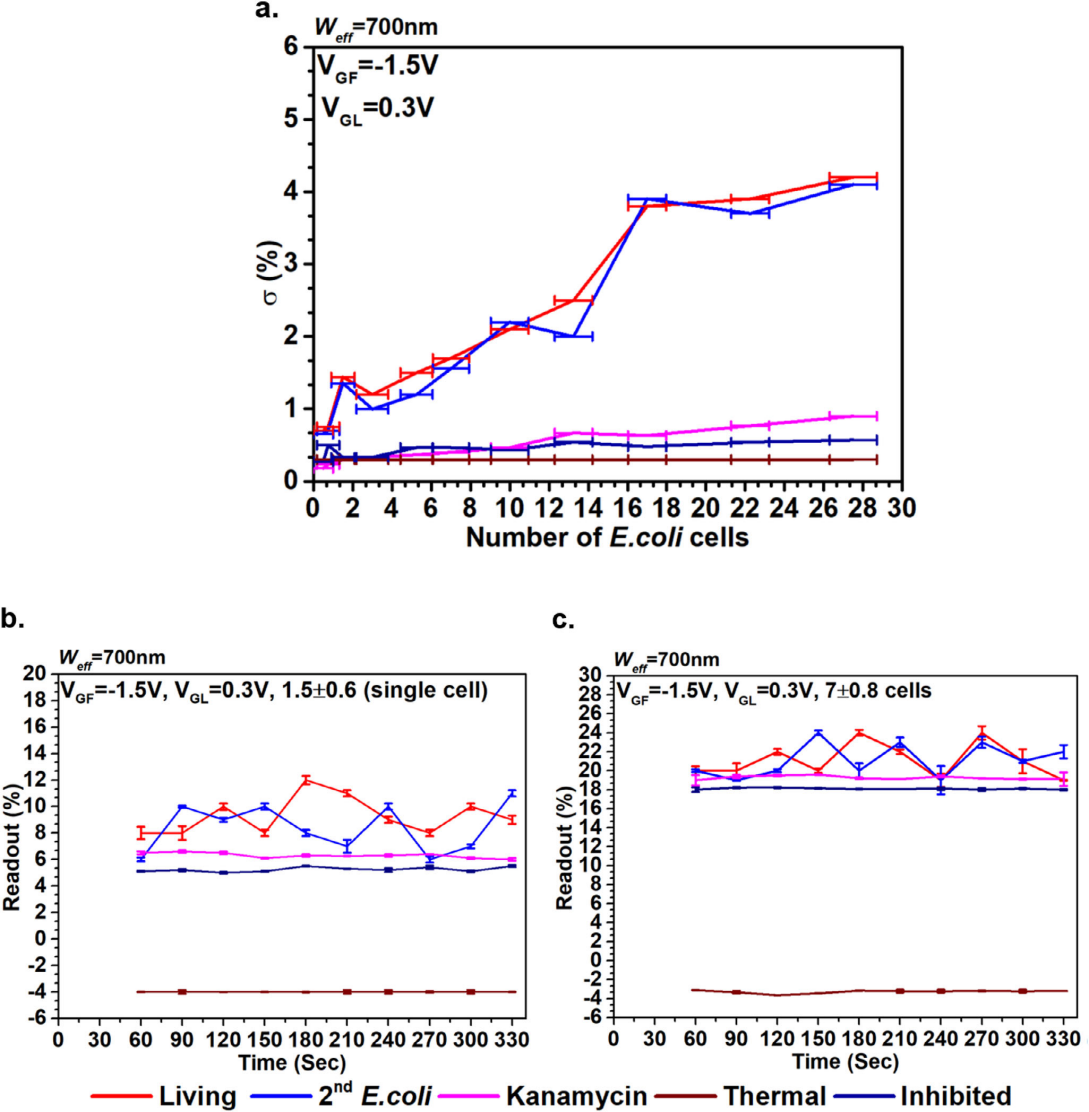
(a) The dependency of σ on living and dead *E. coli* populations at the sensing area. The data points and error bars are averages and standard deviations, respectively, of 10 in‐drop measurements measured across two devices. The measurements are performed in 10 s intervals commencing after 1 min relaxation time. The x‐axis error bars axis reflect the error related to the determination of *E. coli* population in the sensing area (See Methods and Section S4). b. Time‐resolved in‐drop measurements for bacteria population of 1.5±0.6 bacteria. c. Same as b only for bacteria population 7 ± 0.8. The data points and error bars in (b) and (c) reflect measurements performed for two devices.

As stated above and evident in Figure 3, the calibration curves of living and treated *E. coli*, except for the thermally denatured bacteria, are indistinct as anti‐*E. coli* antibodies do not discriminate between living and treated bacteria. However, as hypothesized and presented in the Introduction section, the inherent viability of living bacteria is expected to manifest itself in the process of field‐effect transduction. Specifically, it is expected that living bacteria will present time‐dependent field‐effect transduction. In the current case, this time dependency is measured by recording in‐drop *I_DS_*. Hence, treated bacteria are expected to present low in‐drop variation, consistent with their lack of bacterial motility, metabolic activity, etc. In contrast, it is expected that living bacteria will present higher in‐drop variation, consistent with its activity inducing time‐dependent potential distribution, manifested as an increase in standard deviation of the in‐drop measurements although the detailed transduction mechanism underlying this behavior requires further investigation. This distinction in in‐drop response is evident in Figure 4a presenting the dependency of in‐drop standard deviation (henceforth σ) on *E. coli* population for both living and treated bacteria for nano‐channel configuration presented in Figure 3b. The measurements commence 1 min after relaxation time and are performed every 10 s with a total of 10 in‐drop measurements. These are the same measurements (two devices, *W_eff_* = 700 nm) presented in Figure 3b; in Figure 3 the in‐drop standard deviation is for the first three drops to maintain consistency with the control measurements. In fact, 10 in‐drop measurements are performed as presented in Figure 4. Also, σ values of living *E. coli* increase with *E. coli* population, while σ values of treated *E. coli* show a negligible dependency on *E. coli* population consistent with the static nature of those bacteria. (see Section S9 for more in‐drop populations for different *E. coli* populations and for other nano‐channel configurations). Figure 4b presents time‐resolved in‐drop measurements for bacteria concentrations of 1.5 ± 0.6 and 7 ± 0.8 cells, for both living and treated bacteria, extracted from Figure 4a (see Section S9 for more in‐drop populations for different *E. coli* populations and for other nano‐channel configurations). The data points are the average of two devices, and the error bars are the respective standard deviations. As expected, the time‐resolved in‐drop measurements of living *E. coli* present greater time‐dependent fluctuations compared with the treated *E. coli*. Finally, 3D numerical calculations are performed to assess the effect of motility and charge fluctuations (emulating metabolic activity) on *I_DS_‐V_GL_* curves supporting the effect of these over field‐effect transductions (Section S10). The discrimination performance was further evaluated using receiver operating characteristic (ROC) analysis based on both the %Readout and σ (Section S11).

Finally, Table 1 compares the bacterial sensing performance of the proposed MNC FET with previously reported FET devices. Evidently, most reports demonstrate competitive LOD values for diluted buffer conditions, excluding challenges and concerns related to Debye screening and non‐specific adsorption. In contrast, the MNC FET operates in complex LB medium with a single‐cell LOD using a sub‐microliter sample volume and with a competitive response time. Additionally, the sensing measurements are repeated for a conventional bioFET (SI11) and presented in Table 1 as well. More importantly, the MNC FET is the first FET biosensor to discriminate between living and dead bacteria with a single‐bacterium resolution.

**TABLE 1:** Comparison table presenting sensing of *E.coli* with various FET biosensors. Abbreviations: Carbon Nanotube FET (CNT‐FET), Graphene Oxide (GO), Boron Nitride‐reduced Graphene Oxide (BN‐rGo), Not Reported (N.R).

FET method	Recognition layers	Media	LOD (CFU/ mL)	Linear Range (CFU/mL)	Linearity	Distinguishing living from dead bacteria	Sample volume (µL)	Detection Time	Refs.
CNT‐FET	Aptamer	PBS	50.56	10^2^–10^4^	0.993	No	5	3.3 min	[31]
Graphene FET	Anti‐*E. coli* antibody	water	single *E. coli*	10^3^–10^5^	N.R		1	50 s	[34]
CNT‐FET	Anti‐*S. aureus* antibody	0.01× PBS	150	N.R	0.954		N.R	35 min	[46]
MXene‐ GO FET	Aptamer	serum	30	3–3×10^6^	0.994		N.R	5 min	[30]
Graphene FET	aptamers	PBS	100	10^2^–10^6^	N.R		0.9	1.2 min	[49]
BN‐rGO gel FET	Anti‐*E. coli* antibody	0.1× PBS	10	10–10^8^	0.9		10	<2 min	[38]
Standard bioFET	Anti‐*E. coli* antibody	LB	10 ± 0.82	10 ± 0.82 to 27.5 ± 1.3 cells	0.6		0.5	1.5 min	Current work (Section S11)
MNC FET	Anti‐*E. coli* antibody	LB	single *E. coli*	1 ± 0.6 to 28 ± 1.3 Cells	0.97	Yes	0.5	1.5 min	current work

## 4 Conclusions

The MNC FET allows quantitative and real‐time detection of *E. coli* bacteria in ultra‐small complex samples with high specificity and without the need for labeling, pre‐measurement washing steps, or sample preparation. The challenge of the Debye length, the challenge of specific sensing in the presence of unwashed physisorbed non‐specific species, and the origin of the accumulation of positive surface charge upon specific binding events are discussed. Biosensor‐based discrimination between dead and living bacteria is an unmet need in various application fields and has not previously been demonstrated. The study presents a biosensor method allowing differentiation between living and treated/dead bacteria with single‐bacterium resolution, utilizing signal variability correlated with bacterial viability and biological activity. The MNC FET benefits from CMOS fabrication, allowing scalable, low‐cost production of biochips with embedded capabilities and excellent sensing performance, coupled with a high multiplexing capacity enabled by the miniaturization available in current microelectronics technologies. The low fabrication cost of the MNC biochips is due to the relaxed micro‐scale dimensions that support low‐cost lithography. Furthermore, the ability of the platform to resolve temporal variations in the electrical signal, as demonstrated in this study, provides an additional sensing dimension beyond conventional amplitude‐based measurements. Although the detailed transduction mechanism underlying these observations requires further investigation, this approach may pave the way for future FET‐based biosensors extending beyond traditional detection methods. Hence, the presented technology represents a promising candidate for next‐generation biosensing applications, including point‐of‐care diagnostics and portable microbial monitoring systems.

## Conflicts of Interest

The authors declare no conflicts of interest.

## Supporting information




**Supporting File**: smll74900‐sup‐0001‐SuppMat.docx.

## Data Availability

The data that support the findings of this study are available from the corresponding author upon reasonable request.
