# Supplementary material for: Discriminating Between Living and Dead Bacteria With a Field‐Effect Transistor Based on Nano‐Channels
Source: Small. 2026 Jul 28;22(54):e74900. doi: 10.1002/smll.74900 (PMC13614294; doi:10.1002/smll.74900)
Supplement: Supplementary file 1 [file SMLL-22-e74900-s001.docx]

**Discriminating between living and dead bacteria with a field-effect transistor based on nano-channels**

Bar Chen^1#^, Vijay Garika^1#^, Shubham Babbar^1^, Pooja Verma^1^, Tom Cohen^1^, Kfir Kattav^1^, Fanny Akselrod^1^, Aparnaa Shree Vaijaikumar^2^, Shankar Bhattarai^2^, Sherina Harilal^3^, Evgeny Pikhay^4^, Inna Shechter^4^, Doron Greental^4^, Muhammad Y. Bashouti^3,5^, Izhar Ron^1^, Yakov Roizin^4^, Barak Akabayov^2*^, Gil Shalev^1,5*^

^1^School of Electrical Engineering, Ben-Gurion University of the Negev, Israel

^2^Department of Chemistry and Data Science Research Centre, Ben-Gurion University of the Negev, 8410501, Beer-Sheva, Israel

^3^Department of Solar Energy and Environmental Physics, Swiss Institute for Dryland Environmental and Energy Research, J. Blaustein Institutes for Desert Research, Ben-Gurion University of the Negev, Midreshet Ben-Gurion 8499000, Israel

^4^Tower Semiconductor, PO Box 619, Migdal Haemek, Israel

^5^The Ilse-Katz Institute for Nanoscale Science and Technology, Ben-Gurion University of the Negev, POB 653, Beer-Sheva 8410501, Israel

^#^Equal contributors

^*^Corresponding authors: akabayov@bgu.ac.il, glshalev@bgu.ac.il

# **SI1. Physical characterization of recognition layer and stability measurements:**

Physical characterization is performed with contact angle, ellipsometry, electrochemical impedance spectroscopy (EIS), and atomic force microscopy (AFM) in solution. The samples are composed of degenerated silicon decorated with 5 nm SiO_2_. Four samples are considered for characterization: bare Si/SiO_2_ (henceforth ‘bare’), Si/SiO_2_ post pirhana activation (hencefcorth ‘activated’), Si/SiO_2_ modified with APTMS (henceforth ‘APTMS’), and Si/SiO_2_ post anti-*E. coli* modification (henceforth ‘antibody’)

Contact angle: Each sample is measured at four different locations, with four measurements at each location (Figure 1).

**Figure 1:** Contact angle measurements

Figure 2 provides a summary of ellipsometry (three measurements per sample) and contact angle. The data points and error bars are the averaged values and standard deviations, respectively. The bare SiO_2_ surface exhibits a moderate contact angle (~30°), indicating a partially hydrophilic surface, with a layer thickness of approximately 7.5 nm, consistent with sample fabrication. The contact angle drops to ~15° post activation, suggesting a highly hydrophilic surface. This step shows a near-zero thickness, supporting the notion of a clean surface. The contact angle increases to ~35° post APTMS, indicating a reduced hydrophilicity due to the presence of terminal amine groups (–NH₂). The thickness of the APTMS layer at this stage is ~0.5 nm, consistent with a thin silane monolayer. Finally, after immobilization of anti-*E. coli* antibodies, there is an increase in contact angle (~60°), indicating a highly hydrophobic surface due to the exposure of protein side chains^1^. Simultaneously, the measured layer thickness increases to ~3.5 nm, consistent with the expected size of IgG antibodies immobilized on the surface.

**Figure 2:** Surface characterizations.

EIS measurements are repeated three times for each sample: the sample and solution are removed post measurement, and the measurement is repeated once the sample and fresh solution are back installed. Figure 3 shows the Nyquist capacitance plot (C″ vs. C′) extracted from EIS measurements. Bare SiO_2_ shows low real (C′) and imaginary (C″) capacitances. An increase in both C′ and C″ is measured post APTMS, reflecting the formation of a thin molecular layer. The primary amine groups interact with the surrounding electrolyte, introducing dipolar polarization and increasing dielectric loss, which is evident in the elevated C″. The largest shift is observed after antibody immobilization, where both C′ and C″ reach maximum values. This is consistent with the presence of a dense, heterogeneous protein layer, rich in polar residues and flexible conformational domains, which enhances interfacial capacitance while also increasing dielectric losses due to local relaxation mechanisms and fluctuating dipoles within the antibody structure. These changes validate each functionalization step and underscore the cumulative effect of adding molecular layers on both the charge capacity of the sensing interface^2^.

**Figure 3:** Nyquist capacitance plot presented (C″ vs. C′).

**Atomic force microscopy (AFM) measurements**

AFM measurements were performed on Si/SiO_2_ samples in a 150 mM PBS solution (pH 7.4) to emulate LB pH level and ionic strength. Figure 4a shows AFM images of an unmodified sample (UNMOD) and a sample modified with anti-*E. coli* antibodies using the MNC-FET biosensor biofunctionalization protocol. The corresponding height distributions are presented in Figure 4b. AFM characterization confirms successful surface functionalization with anti-*E. coli* antibodies. The unmodified surface exhibits a relatively narrow height distribution centered below 1 nm, indicating a smooth interface. Following antibody immobilization, numerous protruding nanostructures appear across the surface, and the height distribution shifts toward larger values, extending up to several nanometers. The observed height increase is consistent with the presence of surface-bound antibodies. Notably, the extracted height distribution reveals that most antibodies exhibit heights below 3 nm, which is significantly smaller than the expected dimensions of a fully upright IgG molecule. This observation suggests that the antibodies predominantly adopt a tilted or flat-on orientation on the surface, thereby positioning the antigen-binding region near the sensing interface and supporting the hypothesis that binding events occur within the effective sensing region of the MNC FET.

**Figure 4:** **a.** AFM images of the unmodified (UNMOD) surface and the modified surface with anti-E. coli antibodies. **b.** The respective height histograms.

# **SI2: Growth of living and treated bacteria**

Morphology determination is performed for microscopy validation for the presence of *E. coli*. Table 1 presents the morphological characterization of *E. coli*. Two microscopic staining methods were used: Crystal violet (Merck, C0775, >90%) and Nigrosine (Merck, 198285, >90%). Each of these techniques highlights unique features of the bacterium and enables accurate identification of its shape, size, and arrangement^3,4^. In the Crystal Violet stain, which serves as the primary stain in the Gram staining process, *E. coli* appeared as short, dark-purple rods (bacilli), evenly distributed across the field of view. The Nigrosine stain, a negative staining method that colors the background without penetrating the cell wall, produced very sharp images of *E. coli* appearing as bright, well-defined rods against a dark background. A comparison of the two staining methods clearly indicates that the observed bacterium is indeed *E. coli*, as its characteristic morphology, a short, elongated rod with rounded edges and random arrangement consistently appeared in both stains^5^. As a result, *E. coli* is identified under the microscope based on its morphology alone, without additional staining.

| ***E. coli* & Crystal Violet** | ***E. coli*. & Nigrosine** |
| --- | --- |
|  |  |

**Table 1:** Morphological characterization of *E. coli*. Mag. X100, Oil immersion.

**OD_600_ and colony formation determination.**

**Living *E. coli*.** Table 2 quantifies the number of living *E. coli* (DH5α and BL21) in the samples. Optical microscopy images are provided as well (these will be used later to determine the number of *E. coli* in MNC bioFET sensing area, with the same manner for all samples). Drop plating is performed on sterile Petri dishes containing nutrient-rich agar medium supporting bacterial growth. Serial dilutions are performed to reach required range of Colony Forming Units (CFU). Based on this count, the CFU/mL value were calculated per standard method^4^. Importantly, we use this method to reflects only the cells that were metabolically active and capable of replication, thereby distinguishing living cells from damaged or dead ones^6^.

| ***E. coli* DH5α** | | | |  |
| --- | --- | --- | --- | --- |
| **Petri dishes** | | ***E. coli* microscope pictures** | |  |
| **CFU/mL**=0 | |  | |  |
|  | |  | |  |
| **CFU/mL**=220000 | |  | |  |
|  | |  | |  |
| **CFU/mL**=320000 | |  | |  |
|  | |  | |  |
| **CFU/mL**=500000 | |  | |  |
|  | |  | |  |
| **CFU/mL**=600000 | |  | |  |
|  | |  | |  |
| **CFU/mL**=1600000 | |  | |  |
|  | |  | |  |
| **CFU/mL**=3280000 | |  | |  |
|  | |  | |  |
| **CFU/mL**=10040000 | |  | |  |
|  | |  | |  |
| **CFU/mL**=25600000 | |  | |  |
|  | |  | |  |
| ***E. coli* BL21** | | | | |
|  |  | |  | |
|  |  | |  | |
|  |  | |  | |

**Table 2:** Quantification of living *E. coli* DH5α and living *E. coli* BL21. CFU and microscopy images are provided (arrows indicating location of bacteria). CFU is determined visually. CFU/mL is determined accounting for the respective LB dilution.

Figure 5 shows E. coli DH5α growth curve (OD_600_ vs. CFU/mL).

**Figure 5:** *E. coli* DH5α growth curve (OD_600_ vs. CFU/mL, same conditions and method for all type of bacteria).

***E. coli* post thermal denaturation and kanamycin.** The samples are plated on agar plates to confirm lack of colony growth (Figure 6). Each petri dish contains 4 dilutions (see marked crosses on dishes).

| **Thermal denaturation (heat treatment), 80 ^0^C, 20 min** | | |
| --- | --- | --- |
|  |  |  |
| **Antibiotic treatment (Kanamycin), 100 μg/mL** | | |
|  |  |  |

**Figure 6:** Petri plates with E. coli post thermal denaturation and antibiotic treatment.

***E. coli* post inhibition**. Table 3 shows *E. coli* inhibition optimization with chloramphenicol concentration (mic-like). The validation is performed with OD_600_ measurements.

| **INHIBITOR ASSAY** | **Sample Name** | **Chloramphenicol (µg/mL)** | **LB (mL)** | **OD** |  |  |
| --- | --- | --- | --- | --- | --- | --- |
|  | DH5α- 10µl | 0 | 5 | 0.988 |  |  |
|  |  |  |  |  |  |  |
|  | DH5α- 10µl | 2 | 5 | 0.132 |  |  |
|  |  |  |  |  |  |  |
|  | DH5α- 10µl | 4 | 5 | 0.025 | **MIC** |  |
|  |  |  |  |  |  |  |
|  | DH5α- 10µl | 6 | 5 | 0.026 |  |  |
|  |  |  |  |  |  |  |
|  | DH5α- 10µl | 8 | 5 | 0.015 |  |  |
|  |  |  |  |  |  |  |
|  | DH5α- 10µl | 10 | 5 | 0.005 |  |  |
|  |  |  |  |  |  |  |
|  | DH5α- 10µl | 12 | 5 | 0.007 |  |  |
|  |  |  |  |  |  |  |
|  | DH5α- 10µl | 15 | 5 | 0.006 |  |  |
|  |  |  |  |  |  |  |
|  | DH5α- 10µl | 20 | 5 | 0.011 |  |  |
|  |  |  |  |  |  |  |

**Table 3:** E. coli inhibition optimization with chloramphenicol concentration.

Table 4 shows petri plates with *E. coli* post inhibition. Upon comparison with same dilutions of Table 2, a substantial reduction in the number of colonies formed on the agar plates is recorded for the inhibited bacteria. While the untreated control culture produced many colonies, indicating normal bacterial proliferation and a high division capacity, the treated culture showed a marked decrease in CFU. Since each colony originates from a single bacterium or a small group of bacteria that retain the ability to divide and proliferate, this finding indicates a significant inhibition of bacterial proliferation. These results are consistent with the well-established mechanism of action of chloramphenicol, which inhibits bacterial protein synthesis and thereby reduces cellular growth and division. Accordingly, this population represents a state of reduced biological activity and suppressed growth compared to living bacteria grown under standard culture conditions.

| ***E. coli* inhibition** | | |
| --- | --- | --- |
|  |  |  |
|  |  |  |

**Table 4:** Selected petri plates with E. coli post inhibition.

**Living *M. smegmatis*.** Table 5 presents petri dishes with living *M. smegmatis.*

| ***Mycobacterium smegmatis* (*M*. *smegmatis*)** | | |
| --- | --- | --- |
|  |  |  |
|  |  |  |

**Table 5:** Selected petri plates with living M. smegmatis.

**SI3: Viability test**

**Bacterial viability test:** Bacterial viability was evaluated using a Resazurin Reduction Assay based on bacterial metabolic activity. A 0.01% (w/v) resazurin stock solution was prepared by dissolving 2.5 mg resazurin sodium salt (Sigma-Aldrich, R7017) in 25 mL sterile distilled water, followed by brief vortex mixing until a homogeneous solution was obtained. The solution was subsequently filtered using a 0.22 µm membrane filter and stored at 4 °C under dark conditions until use. Bacterial cultures were grown in LB medium to the mid-logarithmic growth phase. Subsequently, 1 mL of bacterial culture was centrifuged at 6000 rpm for 5 min. The resulting pellet was washed twice with 1× PBS, prepared from PBS tablets (Sigma-Aldrich, P4417), and then resuspended in 1 mL of 1× PBS. The viability assay was performed on a 96-well plate (Wuxi NEST Biotechnology Co., Ltd., 701101). Each well contained 180 µL of bacterial suspension and 20 µL of the resazurin stock solution, resulting in a final assay volume of 200 µL and a final resazurin concentration of approximately 0.001% (w/v). The plate was incubated at 37 °C under dark conditions, and the reduction-mediated color transition of resazurin was monitored every 15 min for up to 60 minutes. Wells containing 1×PBS and resazurin solution only were used as blank controls. Reduction of resazurin by metabolically active bacteria resulted in a color transition from blue to purple or pink. Blue indicated the absence of metabolic activity, purple indicated intermediate metabolic activity, and pink indicated high bacterial viability (see Table 6, The metabolic activity was determined based on the OD measurements relative to the bulk untreated control, rather than by visual inspection alone). Bacterial viability level quantification was determined according to absorbance measurements at 570 nm using a Synergy H1 microplate reader (BioTek, H1).

The background-corrected signal (ΔA) was calculated by subtracting the blank absorbance from the measured sample absorbance, as shown in Figure 7. Living *E. coli* DH5α and *E. coli* BL21 exhibited the highest ΔA values, indicating strong metabolic activity and efficient resazurin reduction. In contrast, the growth-inhibited *E. coli* population displayed an intermediate signal that was substantially lower than that of living bacteria but clearly higher than that of the thermal and kanamycin controls, demonstrating the retention of partial metabolic activity despite impaired growth. Both thermal and kanamycin-treated bacteria exhibited signals close to the background level, consistent with a significant loss of metabolic function. Collectively, these results demonstrate a clear separation between living, growth-inhibited, and kanamycin treated and thermal denatured bacteria, supporting the conclusion that the inhibited bacteria represent a distinct intermediate physiological state rather than a dead population. The bars represent the mean value of the measurements, while the error bars correspond to the standard error of the mean (SEM).

**Figure 7:** The viability and metabolic activity of the different bacterial populations.

|  |  |
| --- | --- |
|  |  |

**Table 6:** The viability assay. Plates after measurements with Synergy H1 microplate reader (BioTek, H1).

**SI4. Calibration of CFU vs. *E. coli* number in MNC bioFET sensing area**

Figure 8 shows the relationship between E. coli bacteria concentrations in the culture (CFU/mL) and the actual number of E. coli bacteria observed on the active area of the MNC-FET. In the experiment, four separate drops from each known E. coli bacteria concentration were placed onto the MNC-FET active area, and the number of E. coli bacteria within this area was counted using an optical microscope (X1000). For each concentration, four independent drops were analyzed. The number of *E. coli* cells was counted in each drop, and the mean value and standard deviation were subsequently calculated.

Cells (nomial number)

| Bacterial concentration (CFU/mL) | AVG |
| --- | --- |
| 220000 | 0.5 |
| 320000 | 0.75 |
| 500000 | 1.5 |
| 600000 | 3 |
| 1600000 | 5.25 |
| 3280000 | 7 |
| 10040000 | 10 |
| 25600000 | 13.25 |
| 81800000 | 17 |
| 138000000 | 22.25 |
| 150000000 | 27.5 |

**Figure 8:** Number of bacteria measured in the MNC-bioFET's sensing area vs. E. coli bacteria in culture (CFU/mL).

# **SI5. Measurements and calculation of ionic strength and Debye length**

The ionic strength of the LB medium was estimated from conductivity measurements performed on a series of LB dilutions. Since the electrical conductivity of a solution is directly related to the concentration and mobility of dissolved ions, conductivity measurements provide a practical approach for estimating ionic strength in complex growth media. The conductivity of each dilution was measured at room temperature using a conductivity meter (Sigma-Aldrich, Ohaus conductivity meter, OH30058731), and the corresponding ionic strength was calculated from the measured conductivity values. Figure 9a shows the correlation between ionic strength (M) and electrical conductivity of the solution. Figure 9b shows the same analysis in the presence of kanamycin. Note the nearly linear fit, as indicated by the high R² value (0.9916), allowing for an accurate measurement of the solution’s ionic strength through conductivity. This linearity means that ionic strength can be directly and simply estimated based on conductivity measurements^7^.

**a.**

**b.**

**Figure 9:** **a.** The correlation between ionic strength (M) and the electrical conductivity of the solution. **b.** Same with kanamycin.

**Debye length calculation:**

Debye Length Calculation with ionic strength of 197 mM, Eq. (1):

$${\left( 1 \right). \lambda}_{L}=\sqrt{\frac{\varepsilon_{r}\varepsilon_{0}K_{B}T}{2e^{2}I}}$$

$$\varepsilon_{r}=58$$

$$\varepsilon_{0}=8.85\cdot{10}^{-12}\frac{C^{2}{\cdot S}^{2}}{Kg\cdot m^{3}}$$

$$K_{B}=1.38\cdot{10}^{-23}\frac{Kg{\cdot m}^{2}}{S^{2}\cdot K}$$

$$T=298 K$$

$$e=1.6{\cdot10}^{-12}C$$

$$I=197 mM=7.5\cdot{10}^{25}\frac{num}{mol}$$

$$N_{A}=6.022\cdot{10}^{23}\frac{num}{mol}$$

$$\lambda_{L}=\sqrt{\frac{58\cdot8.85\cdot{10}^{-12}\frac{C^{2}{\cdot S}^{2}}{Kg\cdot m^{3}}\cdot1.38\cdot{10}^{-23}\frac{Kg{\cdot m}^{2}}{S^{2}\cdot K}\cdot298 K}{2\cdot{(1.6{\cdot10}^{-12})}^{2}C^{2}\cdot7.5\cdot{10}^{25}\frac{num}{mol}}}=7.55\cdot{10}^{-10}m=\boldsymbol{0.755 nm}$$

Calculated the Debye Length for **ionic charges,** Eq. (2):

$${\left( 2 \right). \lambda}_{L}=\sqrt{\frac{\varepsilon_{r}\varepsilon_{0}K_{B}T}{e^{2}\sum_{i} C_{i}\cdot Z_{i}}}$$

$$Z_{i}- ions charges$$

$$\sum_{i} C_{i}\cdot Z_{i}=273.04\cdot{10}^{-3}\frac{mol}{L}=273.04\frac{mol}{m^{3}}\cdot6.022\cdot{10}^{23}\frac{num}{mol}=1.64\cdot{10}^{26}\frac{num}{mol}$$

$$\lambda_{L}=\sqrt{\frac{58\cdot8.85\cdot{10}^{-12}\frac{C^{2}{\cdot S}^{2}}{Kg\cdot m^{3}}\cdot1.38\cdot{10}^{-23}\frac{Kg{\cdot m}^{2}}{S^{2}\cdot K}\cdot298 K}{\left( 1.6{\cdot10}^{-12} \right)^{2}C^{2}\cdot1.64\cdot{10}^{26}\frac{num}{mol}}}=7.09\cdot{10}^{-10}m=\boldsymbol{0.709 nm}$$

$$\lambda_{L}\boldsymbol{=0.709 nm}$$

Debye Length Calculation with ionic strength (PBS,0.15 mM), Eq. (1):

$$\lambda_{L}\approx\boldsymbol{30 nm}$$

Debye Length Calculation with ionic strength of (PBS, 1.5 mM), Eq. (1):

$$\lambda_{L}\approx\boldsymbol{10 nm}$$

Debye Length Calculation with ionic strength of (PBS, 150 mM), Eq. (1):

$$\lambda_{L}<\boldsymbol{1 nm}$$

**Dependency of sensing performance on Debye length**

*E. coli* sensing is pursued in three dilutions of PBS to determine sensing performance dependency on Debye length. First, viability of *E. coli* in the corresponding PBS dilutions is validated, as described next.

To evaluate the effect of ionic strength on bacterial viability, a resazurin reduction assay was performed using *E. coli* cells in LB, PBS 0.15 mM, PBS 1.5 mM, and PBS 150 mM as shown in Figure 10 (see also viability test above). The ΔA was calculated. As shown in the figure, all tested conditions exhibited ΔA values substantially higher than the background level, indicating significant metabolic activity and preservation of bacterial viability throughout the experiment. These results indicate that the bacterial populations maintained substantial metabolic activity across all tested media and that no significant loss of viability occurred because of changing the ionic-strength conditions. The bars represent the mean value of the measurements, while the error bars correspond to the SEM.

**Figure 10:** E. coli viability various PBS dilutions.

Next, *E. coli* in three PBS dilutions is performed. Sensing is performed in the same manner as provided in the manuscript. Figure 11a,b present the corresponding *I_DS_*-*V_GL_* and %Readout curves, respectively. All measurements are performed with two identical transistors (*W_eff_*= 700nm). Figure 11c shows the calibration curve of all the PBS dilutions.

**C.**

**Figure 11:** **a.** *I_DS_* vs*. V_GL_* measurements performed in PBS solutions with concentrations of 0.15 mM, 1.5mM and 150 mM. Measurements are performed with two transistors of the same channel width (*W_eff_*= 700nm). **b.** The corresponding %Readout-*V_GL_* curves. **c.** The respective calibration curves. The dashed lines are the linear fits. In all curves the data points and error bars are the averages and standard deviations of measurements performed for two devices.

# **SI6. Measurements of non-specific proteins**

The LB medium used in this study was Miller’s formulation (10 g/L NaCl, 10 g/L tryptone, 5 g/L yeast extract):

**Table 8:** LB medium

To determine the amount of protein in LB growth medium, a calibration curve was generated using a series of dilutions of a standard protein solution. The absorbance was measured at 595 nm according to the Bradford assay method. This specific wavelength is used because the Coomassie Brilliant Blue dye, which binds to proteins in the assay, undergoes a shift in its absorbance maximum upon binding, resulting in a strong absorbance peak at 595 nm.

Table 9 presents the model information, while Table 10 and Figure 12 present the calibration curve.: a linear relationship between protein concentration (mg/mL) and absorbance, with a high correlation coefficient (R² = 0.9928), indicating a good fit of the data to the regression line. The equation of the line (y = 0.0118x + 21.976) allows for the calculation of protein concentration in unknown samples based on their measured absorbance values. Using this calibration curve, the protein content in the LB medium after incubation with the samples could be quantitatively assessed.

| Software Version | 3.03.14 |
| --- | --- |
| Date | 26/08/2024 |
| Time | 11:04:51 |
| Reader Type: | Synergy H1 |
| Reader Serial Number: | 1710246 |
| Reading Type | Reader |
| **Procedure Details** | |
| Plate Type | 96 WELL PLATE |
| Read | Absorbance Endpoint |
|  | Wavelengths: 595 |
|  | Read Speed: Normal, Delay: 100 msec, Measurements/Data Point: 8 |
| **Results** | |
| Actual Temperature: | 22.4 |

**Table 9:** Data informetion BSA calibration calculations

| **Standard BSA (Bovine serum albumin) concentration** | **Absorbance (595nm)** | **Subtracted file** |
| --- | --- | --- |
| 0 | 0.225 | 0 |
| 0.0025 | 0.292 | 0.067 |
| 0.005 | 0.347 | 0.122 |
| 0.0075 | 0.409 | 0.184 |
| 0.01 | 0.474 | 0.249 |
| 0.0125 | 0.52 | 0.295 |
| 0.015 | 0.548 | 0.323 |
| 0.0175 | 0.618 | 0.393 |

**Table 10:** BSA calibration

**Figure 12:** BSA calibration curve.

Table 11 shows a clear correlation between the optical density (OD) of the *E. coli* cultures and the total protein concentration measured in each sample. As expected, the LB medium alone (without any bacterial growth) showed a very low protein concentration (0.006 mg/mL), serving as a baseline. In the samples containing *E. coli*, protein levels increased as the OD increased. For example, the sample with an OD of 0.06 had a protein concentration of around 0.011 mg/mL, while the sample with an OD of 1.5 reached approximately 0.110 mg/mL. This trend indicates that bacterial growth in the LB medium led to protein production and/or secretion into the medium, and that the Bradford assay successfully detected these differences in protein concentration across the samples.

| **Name** | **Absorbance** | **Subtracted absorbance** | **Total Protein concentration(mg/mL)** |
| --- | --- | --- | --- |
| **LB** | 0.232 | 0.007 | 0.006463 |
| ***E. coli* 0.06 OD** | 0.237 | 0.012 | 0.011463 |
| ***E. coli* 0.7 OD** | 0.287 | 0.062 | 0.061463 |
| ***E. coli* 1.5 OD** | 0.336 | 0.111 | 0.190463 |

**Table 11:** Based on the standard BSA calibration curve protein, protein concentration in LB and bacteria results

**Non-specific adsorption (ferritin) under different ionic strengths**

Ferritin molecules (ABCAM, AB281293, >90%) are used as a study case to evaluate field-effect transduction of non-specific adsorption. To this end, various concentrations of ferritin are introduced to unmodified MNC bioFET, and sensing is performed in the same manner as reported for *E. coli*. The measurements are performed in PBS solutions with ionic strengths of 0.1, 10, and 100 mM in order to evaluate the effect of screening on field-effect transduction of non-specific species. Ferritin samples in the range of 1 fg/mL to 100 μg/mL are succesivly introduced to the MNC bioFET from lowest to highest concentration. The respective *I_DS_-V_GL_* are presented in Figure 13a. Figures 13b,c presents the respective %Readout-*V_GL_* curve for two *V_GF_* values. Each plot presents 12 curves corresponding to the different ferritin concentrations. In all cases, only minimal signal variations were observed, with no dependence on ferritin concentration. Figures 13d,e presents representative response curves for %Readout-ferritin concentrations. In all cases, the curves remained relatively flat and exhibited non-monotonic response with increasing ferritin concentration indicating the absence of sensitivity toward ferritin. Figure 13f presents %Readout vs. ferritin concentrations. Each plot was obtained at a different *V_GL_* value and includes three curves corresponding to the three ionic strengths. No consistent trend with increasing ferritin concentration was observed, and the curves corresponding to different ionic strengths largely overlapped. In summery, field-effect transduction of non-specific species is negligle and do not depend on Debye length.

**Figure13: a.** The respective I_DS_-V_GL_, **b,c** presents the %Readout vs. V_GL_. **d,e** presents %Readout vs. ferritin concentrations. **f.** presents %Readout vs. ferritin concentrations including all PBS dilutions.

**SI7: Control, stability and reproducibility measurements**

This section presents controls performed in the experiment. Figure 14 shows *I_DS_-V_GL_* and *I_GF_-V_GL_* (left) and *I_GF_-V_GL_* (right), where *I_GF_* is solution leakage current. Figure 15 shows *I_DS_-V_GL_* curves for control measurements. Figure 16 shows the corresponding %Readout-*V_GL_* curves. Figure 17 shows the corresponding calibration curves for controls. Figure 18 shows device reproducibility measurements. Figure 19 shows stability measurements for a biofunctionalized MNC bioFET.

**Figure 14:** left: I_DS_-V_GL_ and I_GF_-V_GL_ and right: I_GF_-V_GL_ (only leakage current).

**Figure 15:** I_DS_-V_GL_ curves for control measurements.

**Figure 16:** %Readout-V_GL_ curves for controls.

**Figure 17:** Calibration curves for control measurements

**Fabrication reproducibility**

Figure 18 presents lateral gate threshold voltage (Vth) producing *I_DS_*=10 nA for different values of *W_eff_* (indicated in the figure as Wn). The measurements are performed in dry conditions and *V_GB_*=5 V. A total of 35 devices are measured for each *W_eff_*. The blue line represents the mean *V_GL_* value and the shaded region corresponds to standard deviations. The data presents excellent repeatability and reproducibility of the silicon chip.

**Figure 18:** Lateral gate threshold voltage (Vth) measured for a drain current of 10 nA as a function of W_eff_ (=Wn).

**Storage stability measurements**

To evaluate its suitability for practical diagnostics, the long-term functional stability of the anti-*E. coli* functionalized MNC bioFET was monitored over seven days. The device was stored at 4 °C and characterized daily under ambient conditions. To ensure statistical reproducibility, five distinct aliquots were measured each day, with each aliquot analysed in triplicate. Between testing cycles, the bioFET was returned to 4 °C storage. The resulting longitudinal data, as shown in Figure 19, demonstrate excellent temporal stability and preserved bio-recognition activity, confirming the MNC platform's robustness for multi-day applications.

**Figure 19:** Storage stability measurements

# **SI8: Specific measurements for other conducting channels**

Figure 20 shows the extracted calibration curves for other conducting channels for the specific and label-free detection of the anti-*E. coli*/ *E. coli* interaction.

**Figure 20:** Calibration curves for other conducting channels

# **SI9: Calibration curves and σ dependency on *E. coli* population for other channel configurations (*W_eff_*=900nm)**

**Figure 21:** Left panel: Calibration curves for specific sensing of living E. coli. and dead E. coli for selected channels. The response curves of corresponding control measurements are provided as well. The x-axis error bars reflect the variation in E. coli population at the sensing area. The y-axis error bars reflect in-drop variation. Right panel: The corresponding σ dependency on E. coli sensing area population. The x-axis error bars reflect the variation in E. coli population at the sensing area.

**SI10: Three dimensional (3D) numerical calculations**

The electrical charge of a single *E. coli* DH5α bacterium is estimated based on its outer membrane composition and electrokinetic properties reported for *E. coli* K-12, from which it is derived. The bacterial outer membrane is rich in LPS, with approximately 2-3x10^6^ LPS molecules per cell^8^, each carrying about 4-6 negative charges, leading to an estimated total of ~10^7^ e elementary charges (~10^-12^ C) on the surface^8–11^. Alternatively, electrokinetic measurements such as zeta potential yield an effective surface charge density on the order of σ~10^-2^ C/m^2 12–15^, assuming a typical geometry (length ~2 µm and radius ~0.5 µm)^16,17^, the surface area is ~9x10^-12^ m^2^, resulting in a more conservative total charge of 10^-13^ C. Accordingly, assuming the *E. coli* is surrounded by a layer of counterions (di/monovalent cations) that neutralize its charge, the number of ions required is of the same order, namely~10^6^-10^7^ ions^18–20^, corresponding to a total positive charge of ~10^-13^-10^-12^ C.

3D device simulations are performed using Sentaurus Device, where the Poisson and Continuity equations are solved at each mesh point. The electrolyte is modeled as a symmetric electrolyte represented by a non-degenerate semiconductor with a bandgap of 1.5 eV, a relative permittivity of εᵣ = 80, and an ionic strength of 100 mM. The electron (anion) and hole (cation) concentrations are defined as (n=p=N_A_ C₀ × 10⁻³ cm⁻³), where N_A_ is Avogadro’s number and C₀= 0.1 is ionic concentration in molarity. The device channel length (source-to-drain separation) is 5 µm, while the spacing between the p-type lateral regions is 700 nm. A 5.5 nm-thick gate dielectric layer is employed. The n-type channel is uniformly doped with phosphorus at 0.9×10¹⁷ cm⁻³, whereas the n-type source and drain regions are doped with phosphorus at 1×10¹⁹ cm⁻³. The p-type lateral regions are heavily doped with boron at 2×10²⁰ cm⁻³. The back-gate voltage is fixed at 0 V throughout all simulations. To investigate the influence of *E. coli* bacteria, a charge box is introduced at the gate dielectric–electrolyte interface, positioned midway between the source and drain and centered between the p-type regions. The box has a surface area of 800 nm² (length 2 µm and width 0.4 µm), corresponding to approximately one-tenth of the bacterial surface area^21–24^, and a height of 10 nm. It is assigned the same material properties as the electrolyte, except for a reduced relative permittivity of εᵣ = 6.5 ^21–24^. An interfacial charge density of 0.5x10^13^ e^+^/cm^2^ (where e is the elementary charge) is applied to the box/gate dielectric interface^21–24^. This charge density corresponds to a total positive charge of approximately 40,000 elementary charges (e^+^). Importantly, due to mesh size definition at the interface, the surface charge is located at a volume of 0-2 nm away from the physical interface.

Figure 22a illustrates the calculated MNC FET device. A two-dimensional cross-sectional view between the source and drain is also shown, illustrating the position of the charge varied from the center location to 50 nm and 100 nm toward the left side. Figure 22b represents the drain current (*I_DS_*) vs *V_GL_* curves for *V_GF_* = -1 V under different surface charge conditions. Calculations are performed for two surface charge values at the interface between the charge box and the gate dielectric, corresponding to 0 and 40,000e^+^. To evaluate the sensitivity of the device to charge variations, the surface charge is further decreased and increased by 10% relative to the 40,000e^+^ value. The corresponding *I_DS_* vs *V_GL_* curves are included in Fig. 22b. Additionally, the charge box is displaced by 50 nm and 100 nm toward the left from the center, and the resulting *I_DS_* vs *V_GL_* curves are also shown. The observed shifts in the drain current demonstrate the effect of change in charge magnitude and charge location. Figure 22c shows a column plot of the drain current values shown in Fig. 22b at a fixed lateral gate voltage, *V_GL_* = -2.4 V. Drain current increases for the surface charges 40,000 e^+^ compared to zero charges (0 e^+^). When surface charge decreases by 10%, drain current value decreases whereas 10% increase in the surface charge increases the drain current. Similarly, changing the position of the charge box with 40,000 e^+^ surface charges from center to 50 nm and 100 nm left alters the drain current. These results demonstrate that the MNC-FET exhibits strong sensitivity to both variations in surface charge and lateral movement of charged species at the sensing interface.

Figure 22d shows the *I_DS_*-*V_GL_* curves for surface charge values of 0 and 40,000 e^+^ under different electrolyte ionic strengths (1 mM, 10 mM, and 100 mM). Note that at 1 mM and 10 mM (with Debye lengths of ~9.6 nm and ~3 nm, respectively) the current shift is similar as in both cases the surface charge resides entirely inside the Debye volume. On the other hand, for 100 mM, with a Debye length <1 nm, the surface charge is partially screened as it is distributed inside a volume of 0-2 nm away from the physical interface and hence the smaller induced shift in in current.

Figure 22e illustrates the effect of V_GL_ biasing on the electrical double layer and conduction channel. The left side of the figure shows the channel cross-section under different V_GL_ biases conditions, where increasingly negative gate voltages lead to stronger channel depletion and formation of narrower conduction channel. The right side presents the electron density depth profile corresponding to *V_GL_* = 0 V, -1 V, and -2 V. The results indicate that variations in *V_GL_* significantly affect the channel carrier concentration, while the electrolyte double layer remains essentially unchanged, demonstrating negligible influence of *V_GL_* on the solution-side charge distribution.

**Figure 22: 3D Numerical calculations.** **a.** The calculated structure and cross-sections. **b.** I_DS_-V_GL_ curves for V_GF_ = -1 V under different surface charge conditions. **c.** The respective I_DS_ for a fixed V_GL_. **d.** I_DS_-V_GL_ curves for surface charge values of 0 and 40,000e+ for various electrolyte ionic strengths. **e.** The effect of V_GL_ on the electrical double layer (energy diagram, right), and conduction channel (electron density, left).

**SI11: *E. coli* sensing with conventional inversion-mode bioFET.**

To realize an inversion-mode bioFET, we utilized an alternative operating mode of the MNC device. In this configuration, the lateral p-type degenerate silicon regions serve as the source and drain contacts. One lateral gate is grounded, while the other is used to apply *V_DS_*. A negative *V_GF_* induces a p-type inversion at the Si/SiO_2_ interface (n-type channel volume), enabling hole conduction across the channel. In this configuration, the channel length is defined by the separation between the p-type regions (L=0.7 μm), while the channel width (W) is 10 μm (which corresponds to the channel length of the MNC bioFET). Figure 23a presents the *I_DS_-V_GF_* curve and the corresponding sensor response curve, evaluated for *E. coli* populations identical to those used for the MNC bioFET experiments. To establish the noise level, a rigorous repeatability protocol was followed, consisting of three consecutive measurements across 10 independently applied drops. Based on these measurements, the extracted limit of detection (LOD) is 10 *E. coli*, as shown in the calibration curve, Figure 23b.

**a.**

**b.**

**Figure 23:** **E. coli sensing with conventional hole-conducting inversion-mode BioFET.** **a.** I_DS_-V_GF_ for different E. coli populations. **b.** The corresponding response curve illustrates the dependence of the threshold voltage shift on the E. coli population.

**SI12: Statistical significance testing**

Figure 24 shows correlation analysis demonstrating a strong dependency of the MNC response on bacterial population for the specific E. coli measurements (Pearson r=0.986, Spearman ρ=0.991, R²=0.971), indicating a highly reproducible concentration-dependent sensing behavior. Kanamycin-killed and inhibited bacteria also exhibited strong population-dependent trends, suggesting that the sensor response is influenced by the presence of surface-associated bacterial structures even in the absence of full cellular activity. In contrast, the APTMS, unmodified, and background controls demonstrated substantially lower correlation coefficients and reduced coefficients of determination, indicating weaker and less predictable responses. These results support the conclusion that the specific E. coli measurements provide robust and concentration-dependent sensing performance, while the control groups are dominated by non-specific interactions and background variability.

**Figure 24:** Correlation analysis.

The coefficient of variation (CV) was calculated to evaluate the repeatability and relative variability of the sensor response across the tested bacterial populations. The CV was calculated according to Eq. (2): $CV(\%)=\frac{\sigma}{\mu}\times100$, where $\sigma$is the standard deviation of the repeated measurements and $\mu$is the mean. Unlike the standard deviation alone, the CV normalizes the variability to the signal magnitude and therefore provides a direct measure of measurement precision. As shown in Figure 25, the specific measurement exhibited higher CV values than all control groups. In contrast, the APTMS, unmodified, background, kanamycin-treated, and inhibited controls generally exhibited CV values below 6%, indicating more stable and less dynamic electrical response. The elevated CV observed for the specific *E. coli* measurements is not indicative of poor reproducibility but rather is consistent with the increased temporal fluctuations associated with living bacteria specifically bound to the sensing area. These fluctuations are consistent with the dynamic behavior observed in the time-domain measurements. This observation supports the central hypothesis of this work that living bacteria are associated with time-dependent electrical signatures that can be distinguished from inactive or non-specifically bound bacterial populations.

**Figure 25:** CV analysis.

To further evaluate the statistical significance of the differences observed between the specific measurements and the controls, a Wilcoxon signed-rank test was performed across the entire tested bacterial population range (Figure 26). This non-parametric statistical test compares paired measurements and determines whether one response curve is consistently different from another without assuming a normal distribution of the measurements. The analysis was performed using the mean response values obtained at each bacterial population level. The resulting p-values were transformed to $-log(p)$, where larger values indicate stronger statistical significance. All comparisons yielded statistically significant differences ($p<0.01$), indicating that the response curve of the specific measurements is consistently distinct from those of the controls throughout the tested bacterial population range. These results provide additional statistical support that the response observed in the specific measurements is not attributable to random variation alone but rather reflects a unique sensing behavior that is significantly different from that of the control populations.

**Figure 26:** Wilcoxon signed-rank test.

To quantitatively evaluate the ability of the MNC-FET to discriminate between different bacterial populations, receiver operating characteristic (ROC) analysis was performed using two parameters: the %Readout (Figure 27a,c,e,g,i) and the standard deviation (σ) of the electrical signal (Figure 27b,d,f,h,j). The analysis was performed using the complete dataset across all tested concentrations, with each concentration treated as an independent observation. Consequently, the reported area under the curve (AUC), sensitivity, specificity, and optimal threshold reflect the overall discriminative performance of each parameter across the entire concentration range. ROC curves were generated by calculating the sensitivity and specificity over all possible decision thresholds. The optimal threshold was determined using the Youden index, and the area under the ROC curve (AUC) was used to quantify the overall classification performance, where an AUC of 1.0 indicates perfect discrimination and an AUC of 0.5 corresponds to random classification. When comparing the live bacterial group with all control groups combined (Figure 27a,b), the %Readout yielded an AUC of 0.790, with an optimal threshold of 8.78, 81.8% sensitivity, and 72.7% specificity, indicating good but incomplete discrimination due to overlaps with the kanamycin-treated and inhibitor-treated groups. In contrast, σ achieved an AUC of 0.993, with a threshold of 0.70, 100% sensitivity, and 96.4% specificity, demonstrating near-perfect discrimination. To further evaluate bacterial viability, the kanamycin-treated and heat-shock-treated bacteria were combined into a single dead-bacteria group (Figure 27c,d). The %Readout achieved an AUC of 0.769, with 100% sensitivity but only 54.5% specificity, indicating considerable overlap between live and dead bacteria. In contrast, the σ yielded an AUC of 0.983, 100% sensitivity, and 90.9% specificity, confirming that signal fluctuations provide substantially better discrimination than signal amplitude. When live bacteria were compared only with kanamycin-treated bacteria (Figure 27e,f), the %Readout showed limited discrimination (AUC = 0.537, threshold = 26.0, sensitivity = 36.4%, specificity = 72.7%), whereas the σ provided markedly improved performance (AUC = 0.967, threshold = 1.20, sensitivity = 81.8%, specificity = 100%). A comparison with heat-shock-treated bacteria (Figure 27g,h) resulted in perfect classification for both parameters (AUC = 1.0, 100% sensitivity, 100% specificity), indicating complete separation between the two populations. Finally, comparison with inhibitor-treated bacteria (Figure 27i,j) demonstrated that the %Readout exhibited poor discrimination (AUC = 0.554, threshold = 8.787, sensitivity = 81.8%, specificity = 36.4%), whereas the σ again achieved perfect classification (AUC = 1.0, threshold = 0.70, 100% sensitivity, 100% specificity). Overall, the σ consistently outperformed the %Readout across all comparisons, exhibiting higher AUC values together with superior sensitivity and specificity. These findings indicate that the temporal dynamics of the electrical signal contain additional discriminative information beyond the average signal amplitude, substantially improving the ability of the MNC-FET platform to distinguish between live bacteria, dead bacteria, and metabolically inhibited bacteria. The ROC analysis is presented only as a preliminary, exploratory comparison to illustrate the relative discriminative performance of the evaluated parameters.

| **a.** | **b.** |
| --- | --- |
|  |  |
| **c.** | **d.** |
|  |  |
| **e.** | **f.** |
|  |  |
| **g.** | **h.** |
|  |  |
| **i.** | **j.** |
|  |  |

**Figure 27:** Receiver operating characteristic (ROC) analysis of the MNC-FET response based on %Readout (a, c, e, g, i) and the standard deviation (σ) of the electrical signal (b, d, f, h, j). Panels **(a,b)** compare the live bacterial group with all control groups combined; **(c,d)** compare the live bacterial group with the combined dead-bacteria group (kanamycin-treated and heat-shock-treated bacteria); **(e,f)** compare the live bacterial group with kanamycin-treated bacteria; **(g,h)** compare the live bacterial group with heat-shock-treated bacteria; and **(i,j)** compare the live bacterial group with inhibitor-treated bacteria.

**Reference:**

1.	Danish, M. Contact Angle Studies of Hydrophobic and Hydrophilic Surfaces. in *Handbook of Magnetic Hybrid Nanoalloys and their Nanocomposites* 761–782 (Springer International Publishing, Cham, 2022). doi:10.1007/978-3-030-90948-2_24.

2.	Magar, H. S., Hassan, R. Y. A. & Mulchandani, A. Electrochemical Impedance Spectroscopy (EIS): Principles, Construction, and Biosensing Applications. *Sensors* **21**, 6578 (2021).

3.	World Health Organization & J. Vandepitte. Basic laboratory procedures in clinical bacteriology, 2nd ed. *World Health Organization* **2**, (2003).

4.	World Health Organization & J. Vandepitte. *Basic Laboratory Procedures in Clinical Bacteriology*. (1991).

5.	Reshes, G., Vanounou, S., Fishov, I. & Feingold, M. Cell Shape Dynamics in Escherichia coli. *Biophys. J.* **94**, 251–264 (2008).

6.	Cangelosi, G. A. & Meschke, J. S. Dead or Alive: Molecular Assessment of Microbial Viability. *Appl. Environ. Microbiol.* **80**, 5884–5891 (2014).

7.	Woszczyk, M. *et al.* Empirical Formula to Calculate Ionic Strength of Limnetic and Oligohaline Water on the Basis of Electric Conductivity: Implications for Limnological Monitoring. *Water (Basel).* **15**, 3632 (2023).

8.	Raetz, C. R. H. & Whitfield, C. Lipopolysaccharide Endotoxins. *Annu. Rev. Biochem.* **71**, 635–700 (2002).

9.	Erridge, C., Bennett-Guerrero, E. & Poxton, I. R. Structure and function of lipopolysaccharides. *Microbes Infect.* **4**, 837–851 (2002).

10.	Rietschel, E. T. *et al.* Bacterial endotoxin: molecular relationships of structure to activity and function. *The FASEB Journal* **8**, 217–225 (1994).

11.	Clifton, L. A. *et al.* Asymmetric phospholipid: lipopolysaccharide bilayers; a Gram-negative bacterial outer membrane mimic. *J. R. Soc. Interface* **10**, 20130810 (2013).

12.	Wilhelm, M. J. *et al.* Determination of bacterial surface charge density via saturation of adsorbed ions. *Biophys. J.* **120**, 2461–2470 (2021).

13.	van der Wal, A., Norde, W., Zehnder, A. J. B. & Lyklema, J. Determination of the total charge in the cell walls of Gram-positive bacteria. *Colloids Surf. B Biointerfaces* **9**, 81–100 (1997).

14.	Poortinga, A. T., Bos, R., Norde, W. & Busscher, H. J. Electric double layer interactions in bacterial adhesion to surfaces. *Surf. Sci. Rep.* **47**, 1–32 (2002).

15.	Duval, J. F. L. & O. H. Electrokinetics of soft particles. *Langmuir 22,* **22**, 3533–3546 (2006).

16.	Sezonov, G., Joseleau-Petit, D. & D’Ari, R. Escherichia coli Physiology in Luria-Bertani Broth. *J. Bacteriol.* **189**, 8746–8749 (2007).

17.	Idalia, V.-M. N. & Bernardo, F. Escherichia coli as a Model Organism and Its Application in Biotechnology. in *Escherichia coli Recent Advances on Physiology, Pathogenesis and Biotechnological Applications* (InTech, 2017). doi:10.5772/67306.

18.	Clifton, L. A. *et al.* Effect of Divalent Cation Removal on the Structure of Gram-Negative Bacterial Outer Membrane Models. *Langmuir* **31**, 404–412 (2015).

19.	Israelachvili, J. N. *Intermolecular and Surface Forces*. (2011).

20.	Duval, J. F. L. & Ohshima, H. Electrophoresis of Diffuse Soft Particles. *Langmuir* **22**, 3533–3546 (2006).

21.	An, L. *et al.* Protein Apparent Dielectric Constant and Its Temperature Dependence from Remote Chemical Shift Effects. *J. Am. Chem. Soc.* **136**, 12816–12819 (2014).

22.	Löffler, G., Schreiber, H. & Steinhauser, O. Calculation of the dielectric properties of a protein and its solvent: theory and a case study. *J. Mol. Biol.* **270**, 520–534 (1997).

23.	Mobley, D. L., Dill, K. A. & Chodera, J. D. Treating Entropy and Conformational Changes in Implicit Solvent Simulations of Small Molecules. *J. Phys. Chem. B* **112**, 938–946 (2008).

24.	Li, L., Li, C., Zhang, Z. & Alexov, E. On the Dielectric “Constant” of Proteins: Smooth Dielectric Function for Macromolecular Modeling and Its Implementation in DelPhi. *J. Chem. Theory Comput.* **9**, 2126–2136 (2013).
